# Uncertainty Estimation for Dual View X-ray Mammographic Image Registration Using Deep Ensembles

**DOI:** 10.1007/s10278-024-01244-1

**Published:** 2024-09-23

**Authors:** William C. Walton, Seung-Jun Kim

**Affiliations:** 1 University of Maryland, Baltimore County, CSEE Department, Baltimore, MD 21250 USA; 2 The Johns Hopkins University Applied Physics Laboratory, Laurel, MD 20723 USA

**Keywords:** Uncertainty, Image registration, Neural network, Mammography, Breast cancer, Lesion correspondence

## Abstract

Techniques are developed for generating uncertainty estimates for convolutional neural network (CNN)-based methods for registering the locations of lesions between the craniocaudal (CC) and mediolateral oblique (MLO) mammographic X-ray image views. Multi-view lesion correspondence is an important task that clinicians perform for characterizing lesions during routine mammographic exams. Automated registration tools can aid in this task, yet if the tools also provide confidence estimates, they can be of greater value to clinicians, especially in cases involving dense tissue where lesions may be difficult to see. A set of deep ensemble-based techniques, which leverage a negative log-likelihood (NLL)-based cost function, are implemented for estimating uncertainties. The ensemble architectures involve significant modifications to an existing CNN dual-view lesion registration algorithm. Three architectural designs are evaluated, and different ensemble sizes are compared using various performance metrics. The techniques are tested on synthetic X-ray data, real 2D X-ray data, and slices from real 3D X-ray data. The ensembles generate covariance-based uncertainty ellipses that are correlated with registration accuracy, such that the ellipse sizes can give a clinician an indication of confidence in the mapping between the CC and MLO views. The results also show that the ellipse sizes can aid in improving computer-aided detection (CAD) results by matching CC/MLO lesion detects and reducing false alarms from both views, adding to clinical utility. The uncertainty estimation techniques show promise as a means for aiding clinicians in confidently establishing multi-view lesion correspondence, thereby improving diagnostic capability.

**Supplementary Information:**

The online version contains supplementary material available at 10.1007/s10278-024-01244-1.

## Introduction

Methods for generating uncertainty estimates for a convolutional neural network (CNN) technique which registers dual-view X-ray mammographic images are explored in support of breast cancer detection. Breast cancer is one of the leading causes of death for women globally, with over half-a-million lives lost annually, including over 40,000 in the US alone [1]. X-ray is the primary imaging capability used for annual breast cancer screening exams. Clinicians routinely examine at least two X-ray image views, taken from different angles [2]. This helps the clinician to better localize and characterize potential abnormalities. The most frequently used views are the craniocaudal (CC) view, taken from an angle of 0$^{\circ }$ from the top to the bottom of the compressed breast, and the mediolateral oblique (MLO) view, taken at an angle in the range of 45 to 50$^{\circ }$ from medial, near the center of the chest, toward the axilla [3].

In cases where the breast tissue is dense (i.e., involving significant fibro glandular, versus fatty tissue), it can be difficult to distinguish abnormalities from the surrounding tissue; thus, increasing the possibility that a lesion is missed [4]. Studies also show that a significant portion of missed lesions are detected retrospectively [5]. Thus, automated registration tools, which can quickly provide a mapping of corresponding tissue between the two views, can be beneficial in helping clinicians locate lesions in X-ray images, and in particular, during the original exam.

However, there is limited research on the use of automated registration techniques (especially deep learning-based techniques) for finding lesion correspondences between the CC and MLO X-ray mammography views using only the X-ray images (i.e., without the aid of other modalities or 3D information, which may not be available) [6–11]. Moreover, among the reported tools, the issue of the uncertainty has not been investigated enough.

Uncertainty is defined by the US National Institute of Standards and Technology (NIST) as a second value accompanying a measurement, which quantifies the “doubt” about the measurement [12]. At a minimum, uncertainty may be described quantitatively by indications of the dispersion, such as by a probability distribution or standard deviation [13]. In the medical field, uncertainty estimates can be important to clinicians for decision-making, as they can reveal the degree to which measurements or solutions can be “trusted” [14]. If tools that perform automated CC/MLO lesion correspondence also provide an indication of uncertainty, this would further aid clinicians in localizing and characterizing lesions between the two views with confidence.

In this paper, uncertainty estimation techniques are developed for CC-to-MLO lesion registration, through extensive modifications to a CNN-based registration method, on which we previously reported [8]. To our best knowledge, existing CNN-based lesion correspondence techniques, including ours [8], did not address the uncertainty estimation problem. Several architectural variations are experimented with, including networks that generate uncertainty estimates as a second set of outputs in addition to the primary registration mapping outputs. Deep ensemble approaches are adopted, given their reported strength for both accuracy improvement and uncertainty quantification, as well as their implementation practicality [15]. The uncertainty techniques are evaluated on multiple data sets, including computer-simulated and real X-ray images, using several performance measures. The utility of the uncertainty estimates is also demonstrated in a dual-view lesion detection application.

## Related Works

In recent years, uncertainty estimation has become an important topic for deep learning, and various relevant techniques have been reported across domains [14, 16, 17]. Uncertainty in deep learning can generally be ascribed to two different origins: aleatoric (inherent uncertainty in the data itself due to random or noisy attributes) and epistemic (uncertainty of the model) [18]. Aleatoric uncertainty cannot be reduced by increasing the amount of data, whereas epistemic uncertainty can generally be improved in this manner. Since uncertainty is affected by both data and models, uncertainty estimation techniques are not necessarily independent of either category. Note that different domains (e.g., machine learning versus statistics) may refer to aleatoric and epistemic uncertainty using different nomenclature [18].

A high-level categorization of uncertainty quantification techniques in deep learning includes the following: single network deterministic methods, Bayesian methods, ensemble methods, and test-time augmentation methods [14]. Among these, Bayesian methods, which model network parameters as random samples from distributions, are considered the gold standard [19]. Yet, in practice, they require significant network design modification and are also reported as sensitive to domain shift [15, 20, 21]. Hence, techniques for approximating Bayesian uncertainty, such as Monte Carlo dropout [22] or altogether different techniques, are often used [23].

Among the non-Bayesian techniques, deep ensembles are reported to have a performance comparable to Bayesian techniques. The technique involves training several networks seeded randomly to facilitate independence and then applying them during inference to combine the results into one prediction. In the simple ensemble approach, the variance of the primary predictions of the member networks is taken as the variance estimate of the prediction. Leveraging earlier variance estimation research [24], Ref. [15] reported on a deep ensemble approach in which each member network outputs not only the primary output predictions, but also the variance estimate of the primary prediction. The variance estimates are combined into a single variance for the overall prediction. This dual-output approach often demonstrated superior performance over the simple ensemble approach. It also showed good performance amidst domain shift, outperforming Monte Carlo dropout techniques. Given the excellent performances, we pursue both the simple ensemble and dual-output ensemble approaches for our CNN-based mammographic image registration method.

**Fig. 1:**
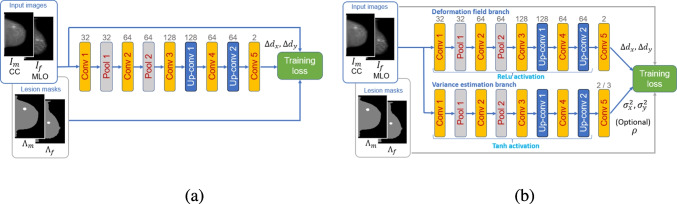
CNN architectures. **a** Serial architecture. **b** Dual-path architecture

A closely related topic to uncertainty estimation is the topic of uncertainty calibration or methods of assessing the quality of uncertainty estimates. Well-calibrated uncertainty values are desired, because if an uncertainty estimate for a network output is uncalibrated, then even when the network indicates that an output has low uncertainty (i.e., high confidence), the quality of the output may actually be poor [25]. To address this, various methods for assessing the quality of uncertainty estimates have been reported [21, 25, 26].

In image registration, uncertainty estimation has long been employed and can serve as a measure of confidence in a registration mapping [27–29]. Registration uncertainty is typically represented as pixel-level error values such as mean squared error, a heatmap across an image, or as ellipses which give indication of uncertainty for various locations.

In machine learning for medical imaging, including in mammography, numerous applications involve uncertainty estimation, such as lesion detection [30], segmentation (e.g., lung or brain tissue) [31], abnormality recognition [32], tissue classification [33–35], image synthesis [36], image registration [17, 37], and other tasks [14, 16]. In mammography, uncertainty is also important for stereotactic procedures such as stereotactic guided biopsy where the minimization of localization uncertainty is important [38, 39]. However, for CNN-based image registration of the CC and MLO X-ray images, to our knowledge, there has been limited research on uncertainty quantification, which is likely due to the limited research on this topic in general.

## Methods

The prescribed methods for uncertainty prediction involve significant extensions to one of our prior CNN registration architectures (which did not provide uncertainty estimates), as discussed in our previous work [8], to which the reader is referred for details, including network design characteristics, performance analysis, and insight into a custom distance-based regularization (DBR) technique. Here, we reformulate DBR for our proposed uncertainty prediction techniques. We commence by restating the registration problem formulation as described in Ref. [8] but with the incorporation of an additional component which represents a new uncertainty output.

### Problem Formulation

A moving image ${I_m}({\varvec{x}})$ and a fixed image ${I_f}({\varvec{x}})$, also referred to as the source and the target images, respectively, are defined with 2D pixel coordinates ${\varvec{x}}\in \Omega \subset \mathbb {R}^2$. The goal is to learn a function $D_{\varvec{\theta }}({I_f},{I_m}) = (d,\Sigma )$, represented by a CNN with parameter vector ${\varvec{\theta }}$, which yields a deformation field $d: \Omega \rightarrow \Omega$ that warps the moving image to match the fixed one, and also a covariance field $\Sigma : \Omega \rightarrow \mathbb {S}_+^2$, where $\mathbb {S}_+^2$ is a set of $2 \times 2$ positive semidefinite matrices, defined for each ${\varvec{x}}\in \Omega$. (This is distinct from the prior formulation, which did not have uncertainty estimates, thus, $D_{\varvec{\theta }}({I_f},{I_m}) = d$). In essence, it is desired that $({I_m}\circ d)({\varvec{x}})$ is similar to ${I_f}({\varvec{x}})$ and that $\Sigma ({\varvec{x}})$ is indicative of the covariance (i.e., uncertainty) associated with each vector mapping. Optimization of the overall mapping is facilitated by a loss function involving a suitable similarity measure *S* and regularizer $R(D_{\varvec{\theta }})$, which govern the nature of the resulting deformation field based on prior knowledge. Metrics related to uncertainty estimation will also be captured in *R*. The general form of the loss function is then defined as

1 $$\begin{aligned} L(I_f,I_m) := -S({I_f}, {I_m}\circ d) + \lambda R(D_{\varvec{\theta }}(I_f,I_m)) \end{aligned}$$

where $\lambda \ge 0$ is a weight to balance the similarity and the regularization terms. The CNN training amounts to solving

2 $$\begin{aligned} \min _{\varvec{\theta }}\ \mathbb {E}_{{\mathcal {D}}}\{L(I_f,I_m)\} \end{aligned}$$

where $\mathbb {E}_{\mathcal {D}}\{\cdot \}$ represents taking an average with respect to the data set ${\mathcal {D}}$ of the fixed and moving image pairs $({I_f},{I_m})$.

It is emphasized that although (1) is formulated to obtain a pixel-wise mapping $d({\varvec{x}})$ for each image pair, our goal is not so much to achieve precise pixel-level registration, as is to establish a useful correspondence between regions of interest, such as lesions.^1^ Further, $\Sigma ({\varvec{x}})$ is desired to serve as, or support the generation of, a useful uncertainty for the mapping. Hence, our objective is that when a clinician selects a candidate lesion location ${\varvec{x}}$ in one view, the trained network can present a candidate location $d({\varvec{x}})$, with an uncertainty quantification $\Sigma ({\varvec{x}})$, for the lesion in the other view.

**Fig. 2:**
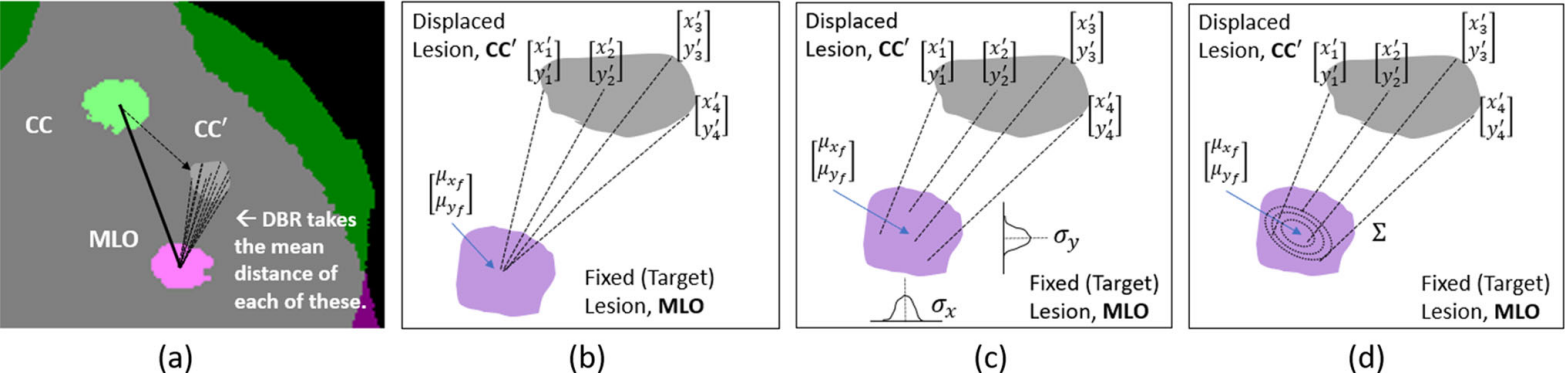
**a** Lesion positions before and after registration. The CC and the MLO views are superimposed, and the ground truth lesion masks in the CC/MLO views, as well as the warped CC lesion (marked as CC$'$), are indicated. **b** Distances between displaced CC lesion pixels ${\varvec{x}}' = [x',y']^T:= d({\varvec{x}})$ and the MLO lesion centroid. **c** DBR with horizontal and vertical component variances of displacements from lesion centroid. **d** DBR with covariance of displacements from lesion centroid

### Network Architectures

Figure 1a shows our original serial CNN architecture reported in Ref. [8]. Figure 1b shows a significant modification to this network, involving dual paths, in which the second path is used to provide variance estimates, $\sigma ^2_x$ and $\sigma ^2_y$, representing uncertainties for the horizontal and vertical deformation components. Here, the horizontal and vertical deformations are treated as uncorrelated. However, a further modification to Fig. 1b (representing yet a third network variation) also yields correlation coefficient estimates, $\rho$, thus estimating parameters for a full covariance matrix. Thus, we have three variations of a fully convolutional neural network (FCN), which we will refer to as Networks 1, 2, and 3, respectively in subsequent discussion.

The serial architecture in Fig. 1a contains five convolution layers, two pooling layers, and two up-convolution layers. The final layer results in two feature maps that correspond to the horizontal and vertical displacements for each pixel, $\Delta d_x$ and $\Delta d_y$ (together constituting $\Delta d({\varvec{x}})$), where ${\varvec{x}}=[x,y]^T$, with $^T$ denoting transposition. The numbers on top of each layer represent the number of feature maps at the output of the layer. The convolution layers also include batch normalization and nonlinear activation using rectified linear units (ReLUs), except for the final layer, which does not include an activation. The kernel sizes and strides for each layer depend on the input image size, as discussed in Ref. [8]

The dual-path architecture, in Fig. 1b, has an upper branch that is identical to that of the serial architecture. The lower branch involves the same layer components, but with the following distinctions. A hyperbolic tangent activation (tanh) function is used for all convolution layers, other than the final layer. For Networks 2 and 3, a softplus activation, $\hat{{\varvec{z}}} = \log (1 + e^{\varvec{z}})$, where ${\varvec{z}}$ denotes an activation input, is used in the final convolution layer to ensure that variance estimates are nonnegative. For Network 3, a tanh activation, $\hat{\hat{{\varvec{z}}}} = (e^{\varvec{z}}-e^{-{\varvec{z}}})/(e^{\varvec{z}}+e^{-{\varvec{z}}})$, is also used in the final convolution layer to bound $-1 \le \rho \le 1$.

The input to each architecture is the pair of CC/MLO images, $I_f$ and $I_m$, which are input as two channels. A second set of inputs, used only during training, involves lesion location masks which are used in the loss function to support custom training regularization (discussed in Ref. [8]) and variance prediction-related modifications of the loss function, as discussed shortly.

The output of Network 1 is $\Delta d({\varvec{x}})$, which captures the *relative* displacement of pixel ${\varvec{x}}$ (i.e., displacement from its original position to its new position) in the moving image. The outputs of Network 2 are $\Delta d({\varvec{x}})$, $\sigma ^2_x$, and $\sigma ^2_y$, the latter two being the horizontal and vertical variance estimates (i.e., uncertainties) for $\Delta d({\varvec{x}})$. Correspondingly, the outputs of Network 3 are $\Delta d({\varvec{x}})$, $\sigma ^2_x$, $\sigma ^2_y$, and $\rho$. The output deformation field is given as

3 $$\begin{aligned} d({\varvec{x}}) := {\varvec{x}}+ \Delta d({\varvec{x}}). \end{aligned}$$

During training, only the displacements arriving at the pixels within the image boundary are actually employed. Further, the mapping may displace some pixels to the same point.

### Distance-Based Regularization for Network 1 (Prior Network)

In our original work, it was reported that a custom DBR technique significantly aided the registration networks in learning the mapping between lesions in the CC and MLO views [8]. As a form of weakly supervised training [40], DBR incorporates the use of the ground truth locations of lesions in the CC and MLO views, obtained from the pair of binary masks that are provided as additional inputs as discussed in Sect. Network Architectures, and as shown in Fig. 1. A form of DBR is illustrated in Fig. 2a, where lesion positions can be seen on an overlay of the CC and MLO masks. The displaced (warped) CC lesion is denoted as CC$'$. The distances between the displaced CC lesion pixels and the MLO lesion centroid, illustrated in Fig. 2b, are used to compute a distance measure which the network uses as a penalty during the training process. Thus, the penalty is decreased as the network moves the CC pixels closer to the MLO centroid during training. DBR was shown to contribute to the network learning process far more than intensity-based similarity measures and deformation-smoothening regularization techniques in the loss function.

The DBR is further leveraged and expanded in this work for uncertainty quantification. To aid in presenting the expanded versions, we first review the DBR formulation, as presented in Ref. 8, but with minor notation changes. Referring to Fig. 2 a and b, let $\Lambda _f^{(n)}: \Omega \rightarrow \{1,0\}$ be the mask image that has the pixel intensity of 1 within the *n*-th lesion, and 0 outside, in the fixed view ${I_f}$. In the corresponding moving image ${I_m}$, $\Lambda _m^{(n)}: \Omega \rightarrow \{1,0\}$ represents the corresponding lesion mask. Then, let $|\Lambda |:= \sum _{{\varvec{x}}\in \Omega } \Lambda ({\varvec{x}})$ be the number of lesion pixels in a mask $\Lambda$. The centroid $\mu (\Lambda ) \in \mathbb {R}^2$ of a mask $\Lambda$ is defined as

4 $$\begin{aligned} \mu (\Lambda ) := \frac{1}{|\Lambda |} \sum _{\{{\varvec{x}}: \Lambda ({\varvec{x}}) = 1\}} {\varvec{x}}. \end{aligned}$$

Then, the DBR function is defined as

5 $$\begin{aligned} R_{DBR}(d; \{\Lambda _f^{(n)}, \Lambda _m^{(n)}\}) = \frac{1}{N} \sum _{n=1}^N \frac{|\Lambda _m^{(n)}|^{-1} \sum _{\{{\varvec{x}}: \Lambda _m^{(n)}({\varvec{x}}) = 1\}}\Vert \mu (\Lambda _f^{(n)}) - d({\varvec{x}})\Vert _1}{\Vert \mu (\Lambda _f^{(n)}) - \mu (\Lambda _m^{(n)})\Vert _1} \end{aligned}$$

where *N* is the number of lesions in the given image pair $({I_f},{I_m})$. As noted in Fig. 2b, ${\varvec{x}}' = [x',y']^T:= d({\varvec{x}})$, where $[x',y']$ are used to more conveniently express Cartesian coordinates. In general, there can be zero, one, or more annotated lesions in $({I_f},{I_m})$. The regularization function is simply set to zero if there are no lesions annotated. In our work, only images with single lesions are used (i.e., $N=1$). Therefore, we will subsequently suppress the lesion index $^{(n)}$. The numerator of Eq. 5 averages the distances between the centroid of the lesion in the fixed view and the individual pixels in the moving view after the warping is done according to *d*. Hence, the objective is to map the CC lesion pixels towards the centroid region of the MLO lesion.

With DBR as the regularizer, the loss function in Eq. 1 has the form

6 $$\begin{aligned} L({I_f},{I_m}) =&-S({I_f},{I_m}\circ d) + \beta R_{DBR}(d; \{\Lambda _f, \Lambda _m\}) \end{aligned}$$

where $\beta$ is a nonnegative weight for balancing the regularization term with the similarity metric. For the similarity measure, *S*, normalized cross-correlation (NCC) is used based on findings in Ref. [8]. This loss function and DBR regularizer are used for the serial architecture (Network 1).

### Regularization Modifications for Uncertainty Estimation (Networks 2 and 3)

For Networks 2 and 3, DBR is modified to support the generation of the variance-related estimates (i.e., uncertainties) for the displacements. The negative log-likelihood (NLL) of the Gaussian distribution can be used in a neural network cost function to allow the network to learn both mean and variance estimates for a target [15, 24]. Since DBR is the component that influences the learning significantly in our loss function (1), NLL was incorporated into the DBR. (Approaches involving using NLL as a similarity measure were found not to perform well.) This involved substituting a NLL-based term into the numerator of Eq. 5.

For Network 2, the NLL is implemented component-wise. Recalling that ${\varvec{x}}' = [x',y']^T:= d({\varvec{x}})$ and referring Fig. 2c, the horizontal component of NLL is

7 $$\begin{aligned} NLL_x({\varvec{x}}') = \frac{1}{2} \log 2\pi + \frac{1}{2} \log \sigma ^2_x({\varvec{x}}') + \frac{(x' - \mu _{xf})^2 }{2\sigma ^2_x({\varvec{x}}')} \end{aligned}$$

where $\mu _{xf}$ is the horizontal component of the MLO lesion centroid (that is, $[\mu _{xf}, \mu _{yf}]^T:= \mu (\Lambda _f)$), and $\sigma ^2_x({\varvec{x}}')>0$ is the horizontal component variance for the pixel location ${\varvec{x}}'$. The average of $NLL_x$ is computed as

8 $$\begin{aligned} \overline{NLL}_x = \frac{1}{|\Lambda _m|} \sum _{\{{\varvec{x}}: \Lambda _m({\varvec{x}}) = 1\}} NLL_x(d({\varvec{x}})). \end{aligned}$$

The average of the vertical component $\overline{NLL}_y$ is defined likewise. The modified DBR function used in Network 2 is then given as the normalized sum of the two NLL component averages.

9 $$\begin{aligned} R_{DBR2}(D_\theta ; \{\Lambda _f, \Lambda _m\}) = \frac{\overline{NLL}_x + \overline{NLL}_y}{\Vert \mu (\Lambda _f) - \mu (\Lambda _m)\Vert _2}. \end{aligned}$$

For Network 3, referring to Fig. 2d, the bi-variate NLL is employed as

10 $$\begin{aligned} NLL({\varvec{x}}')&= \log (2\pi )+\log (\sigma _x({\varvec{x}}'))+\log (\sigma _y({\varvec{x}}'))+\frac{1}{2}\log (1-\rho ({\varvec{x}}')^2) \nonumber \\&+ \frac{(x' - \mu _{xf})^2}{2(1-\rho ({\varvec{x}}')^2)\sigma ^2_x({\varvec{x}}')} + \frac{(y' - \mu _{yf})^2}{2(1-\rho ({\varvec{x}}')^2)\sigma ^2_y({\varvec{x}}')} \nonumber \\&- \frac{\rho ({\varvec{x}}')}{1-\rho ({\varvec{x}}')^2}\left( \frac{x' - \mu _{xf}}{\sigma _x({\varvec{x}}')}\right) \left( \frac{y' - \mu _{yf}}{\sigma _y({\varvec{x}}')}\right) . \end{aligned}$$

The associated DBR term is given by

11 $$\begin{aligned} R_{DBR3}(D_\theta ; \{\Lambda _f, \Lambda _m\}) = \frac{|\Lambda _m|^{-1} \sum _{\{{\varvec{x}}: \Lambda _m({\varvec{x}}) = 1\}} NLL(d({\varvec{x}}))}{\Vert \mu (\Lambda _f) - \mu (\Lambda _m)\Vert _2}. \end{aligned}$$

### Network Ensembles

**Fig. 3:**
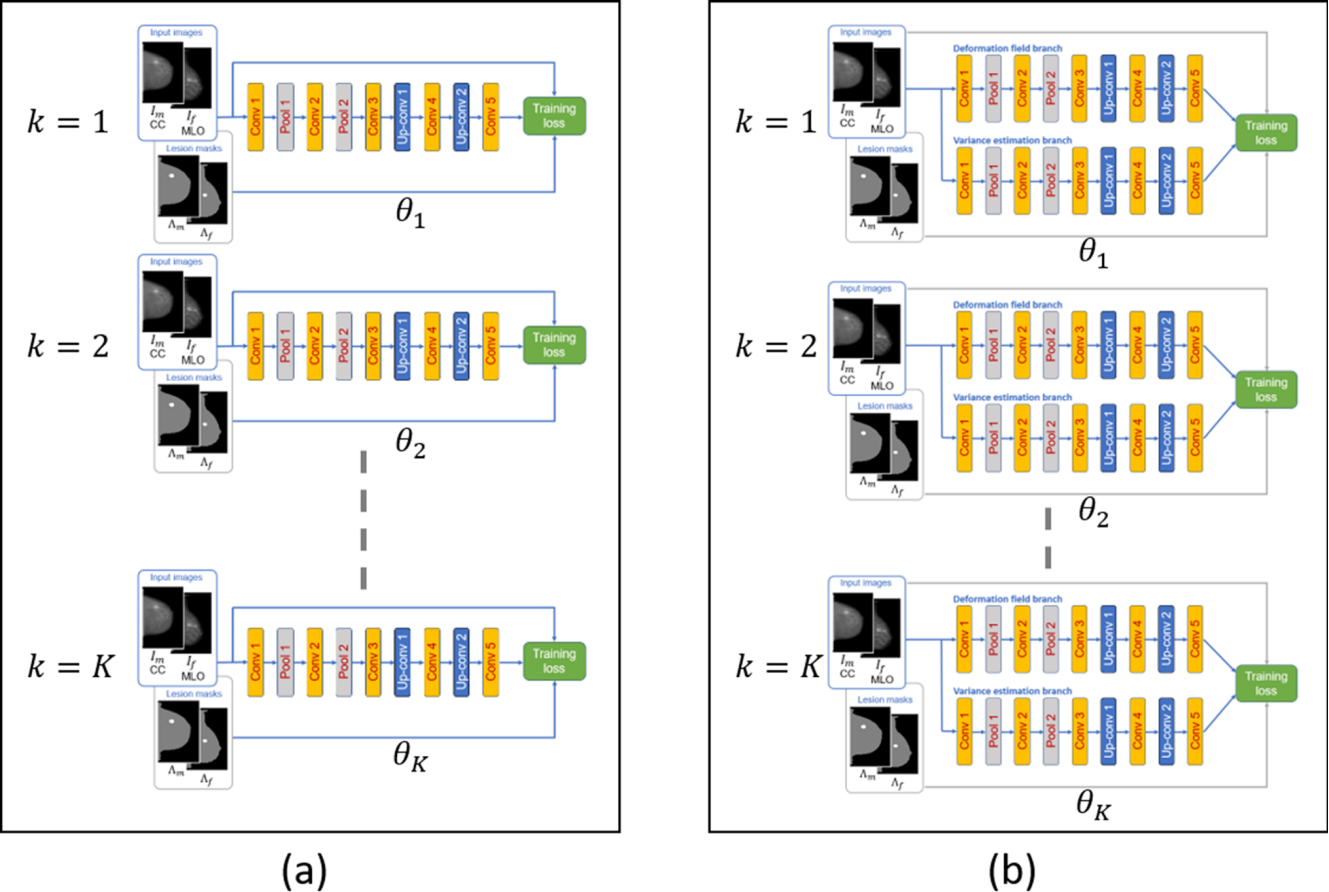
Ensemble architectures (during inference). **a** Simple ensemble involving multiple trained instances of serial architecture. **b** Covariance prediction ensemble involving multiple trained instances of dual-path architecture

During inference, the networks, with respective regularization techniques, described in Section “Network Architectures”–“Regularization Modifications for Uncertainty Estimation (Networks 2 and 3)” are further implemented as ensembles. Referring to Fig. 3a, using Network 1, a simple ensemble is formed by training *K* networks, independently, with random weight initialization. Let $\Delta {d}^{(k)}({\varvec{x}})$ be the displacement at pixel, ${\varvec{x}}$, computed by the *k*-th network. Then, by averaging the displacements from all networks in the ensemble, a mean displacement $\Delta \hat{d}({\varvec{x}})$ is computed along with the estimated covariance $\hat{\Sigma }({\varvec{x}})$ as follows:

12 $$\begin{aligned} \Delta \hat{d}({\varvec{x}})&:= \frac{1}{K}\sum _{k=1}^K \Delta d^{(k)}({\varvec{x}}) \end{aligned}$$

13 $$\begin{aligned} \hat{\Sigma }({\varvec{x}})&:= \frac{1}{K-1}\sum _{k=1}^K \left[ \Delta d^{(k)}({\varvec{x}}) - \Delta \hat{d}({\varvec{x}})\right] \left[ \Delta d^{(k)}({\varvec{x}}) - \Delta \hat{d}({\varvec{x}}) \right] ^T \end{aligned}$$

For Networks 2 and 3, each network in the ensemble produces the parameters for a Gaussian distribution. Thus, the overall estimates can be computed by treating the ensemble as representing a uniformly weighted mixture of Gaussian distribution [15]. For Network 2, letting $\Delta {d}^{(k)}({\varvec{x}}):= [\Delta d_x^{(k)}({\varvec{x}}),\Delta d_y^{(k)}({\varvec{x}})]^T$ and denoting the variance estimates for the horizontal and vertical components from the *k*-th network as $\sigma ^{2(k)}_x({\varvec{x}})$ and $\sigma ^{2(k)}_y({\varvec{x}})$, respectively, the mean horizontal displacement and its variance estimate at pixel ${\varvec{x}}$ are computed as

14 $$\begin{aligned} \Delta \hat{d}_{x}({\varvec{x}})&= \frac{1}{K}\sum _{k=1}^K \Delta d_x^{(k)}({\varvec{x}})\end{aligned}$$

15 $$\begin{aligned} \hat{\sigma }^2_{x}({\varvec{x}})&= \frac{1}{K} \sum _{k=1}^K \{\sigma ^{2(k)}_x + [\Delta d_x^{(k)}({\varvec{x}})]^2 \} - [\Delta \hat{d}_{x}({\varvec{x}})]^2 \end{aligned}$$

respectively. The vertical counterparts $\Delta \hat{d}_y({\varvec{x}})$ and $\hat{\sigma }_y^2({\varvec{x}})$ can be obtained in the same way.

For Network 3, upon denoting the covariance estimate from the *k*-th network as $\Sigma ^{(k)}({\varvec{x}})$, $\Delta \hat{d}({\varvec{x}})$ is again computed as (12), while the ensemble covariance is obtained as

16 $$\begin{aligned} \hat{\Sigma }({\varvec{x}})= \frac{1}{K} \sum _{k=1}^K \left[ \Sigma ^{(k)}({\varvec{x}}) + \Delta {d}^{(k)}({\varvec{x}})\Delta {d}^{(k)}({\varvec{x}})^T\right] - \Delta \hat{d}({\varvec{x}})\Delta \hat{d}({\varvec{x}})^T. \end{aligned}$$

### Spatial Filtering of Estimates

**Fig. 4:**
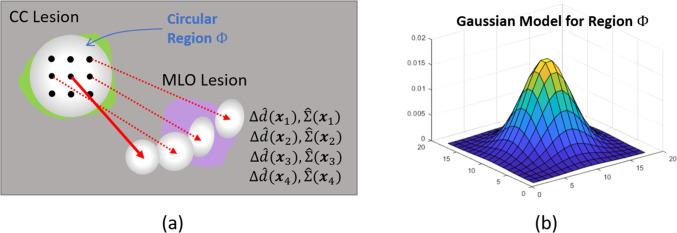
**a** Illustration of a case where a deformation vector emanating from a CC lesion is oriented away from the target MLO lesion whereas neighboring vectors point toward the MLO lesion. **b** A truncated 2D Gaussian kernel is employed to spatially filter the displacements, thus yielding more robust estimates

As discussed in Section “Network Ensembles”, the displacement vector $\Delta \hat{d}({\varvec{x}})$ and the covariance estimate $\hat{\Sigma }({\varvec{x}})$ are obtained based on ensembles for Networks 1, 2, and 3, for each pixel ${\varvec{x}}\in \Omega$. For Network 2, $\hat{\Sigma }({\varvec{x}})$ is defined as the diagonal matrix with $\hat{\sigma }^2_x({\varvec{x}})$ and $\hat{\sigma }^2_y({\varvec{x}})$ on the diagonal. Our preliminary testing of the ensemble estimates and careful observations on the deformation patterns revealed that in some cases, a deformation vector, even one emanating from a lesion, may be oriented quite differently than neighboring vectors. For instance, a given vector may displace a CC pixel away from the target MLO lesion, while neighboring vectors point towards the MLO lesion. An illustration is given in Fig. 4a. In order to reduce such aberrations, a local spatial filtering is applied to the estimates. As there are numerous pixels in lesions, such filtering provides more robust estimates of the displacement and uncertainty, as is validated in our experiments.

Specifically, given a known CC lesion position, a circular region $\Phi ({\varvec{x}}):=\{\bar{{\varvec{x}}}: \Vert {\varvec{x}}- \bar{{\varvec{x}}}\Vert _2 \le R\}$ is defined over the lesion as depicted in Fig. 4a. A 2D Gaussian distribution $\omega (\bar{{\varvec{x}}})$ centered around ${\varvec{x}}$ and truncated to $\Phi ({\varvec{x}})$ as depicted in Fig. 4b is then considered to take a weighted average of $\Delta \hat{d}({\varvec{x}})$ and covariances $\hat{\Sigma }({\varvec{x}})$. In our implementation, the radius *R* and the variance of the Gaussian distribution are fixed based on a heuristic search. The resulting displacement and covariance are computed as

17 $$\begin{aligned} \Delta \bar{d}({\varvec{x}})&= \gamma ({\varvec{x}})^{-1} \sum _{\bar{{\varvec{x}}} \in \Phi ({\varvec{x}})} \omega (\bar{{\varvec{x}}}) \Delta \hat{d}(\bar{{\varvec{x}}}) \end{aligned}$$

18 $$\begin{aligned} \bar{\Sigma }({\varvec{x}})&= \gamma ({\varvec{x}})^{-1} \sum _{\bar{{\varvec{x}}} \in \Phi ({\varvec{x}})} \omega (\bar{{\varvec{x}}}) \left[ \hat{\Sigma }(\bar{{\varvec{x}}}) + \Delta \hat{d}(\bar{{\varvec{x}}})\Delta \hat{d}(\bar{{\varvec{x}}})^T \right] - \Delta \bar{d}({\varvec{x}}) \Delta \bar{d}({\varvec{x}})^T \end{aligned}$$

respectively, where $\gamma ({\varvec{x}}):= \sum _{\bar{{\varvec{x}}} \in \Phi ({\varvec{x}})} \omega (\bar{{\varvec{x}}})$ is the normalization.

Additional details and motivations for spatial filtering are presented in App. A in the Supplementary Material.

## Experiments and Results

### Experiment Setup

#### Data Sets

Three X-ray image data sets were utilized in our experiments as shown in Table 1. The first is a set of synthetic mammogram images generated using software tools developed under the US Food and Drug Administration’s (FDA) Virtual Imaging Clinical Trial for Regulatory Evaluation (VICTRE) project [41]. These synthetic X-ray images were generated from randomly generated in silico 3D phantoms, which simulated physical compression of the breast, different imaging angles, and insertion of lesions at user-prescribed locations, all within certain constraints [41].

The second is the Curated Breast Imaging Subset of the Digital Database for Screening Mammography (CBIS-DDSM), a publicly available set of digitized scanned-film mammography data, curated by trained mammographers [42]. The data set includes the CC and MLO X-ray image pairs for each breast and corresponding binary image masks which indicate the lesion locations.

**Table 1:** X-ray data attributes

	Units	FDA VICTRE	CBIS-DDSM	JHM/TCIA DBT
Image types		Computer	Digitized-	Slices from
		simulated	scanned film (2D)	DBT (2D)
Avg. orig. image size	(Pixels)	2000 $\times$ 1500	5280 $\times$ 3131	2457 $\times$ 1975
Orig. image resolution	($\mu$m)	76	n/a^1^	70
Num. training pairs	(Pairs)	5000	496	152
Num. augmentations		−	8	2 aug &
				5 slices ea.^2^
Total training images	(Pairs)	5000	4464	2280
Num. validation images	(Pairs)	500	−	−
Num. test images	(Pairs)	500	146	258 / 20^3^
Lesion size (Test data)^4^				
Mean	(Pixels)	25.2	20.9	34.3
Std	(Pixels)	0.2	8.0	19.8
Min	(Pixels)	21.9	8.5	9.9
Max	(Pixels)	25.4	46.6	116.4

^1^Image resolution was not available for the CBIS-DDMS data

^2^5 slices were used from each DBT cube. Each slice was augmented 2 times

^3^258 DBT test pairs were used in experiments in Sect. 4.3 and 20 in Sect. 4.4

^4^Lesion size is expressed as diameter in pixels (at the $330 \times 220$ image size) since image pixel resolutions in $\mu$m are not available for all data

The third data set consists of a limited number of de-identified digital breast tomosynthesis (DBT) images with accompanying lesion location information. These were obtained from two sources: a research effort in Johns Hopkins Medicine (JHM) (IRB00185772, 12/3/2018) and a publicly available DBT data set from The Cancer Imaging Archive (TCIA) [43].

Each data set involved only mass-type lesions. Further, only cases with a single mass that was present in both the CC and MLO view were considered due to the lack of ground truth information on lesion correspondence for multiple lesions.

#### Preprocessing

Prior to ingestion into the networks, the images were subjected to several preprocessing steps. These involved reorienting all images to a chest-left orientation, removing certain scene artifacts, extracting the breast tissue region, masking out the pectoral muscle in MLO images, and applying slight rotations for augmentation purposes. The pixel intensities were also normalized to the range of [0, 1]. All images were resampled to a resolution of $330 \times 220$, as this resolution can give indications of performance of higher resolutions, while expediting computing resource utilization, as discussed in Ref. [8] where resolutions ranging from $330 \times 220$ to $990 \times 660$ were tested. For the DBT data, 5 slices, that intersected the lesion, in each cube were used. Also, a subset of DBT test data, which was exclusive to one experiment, involved only 20 test pairs.

**Fig. 5:**
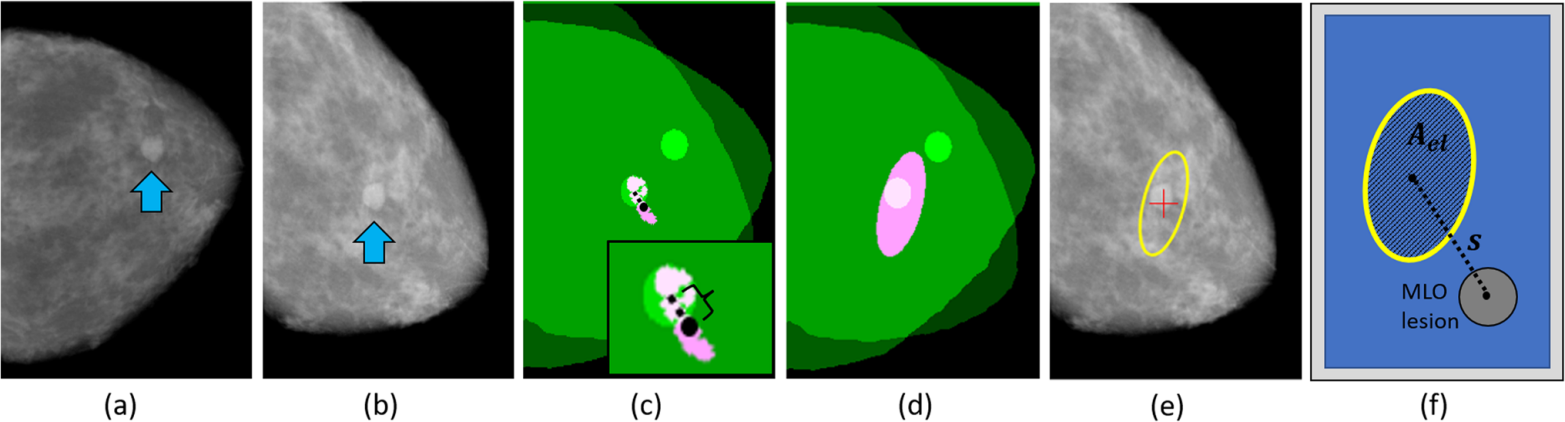
**a** Input CC view with a lesion. **b** Input MLO view with a lesion. **c** Distance between centroids of displaced CC pixels, in magenta, and MLO lesion (on CC/MLO overlay). The large dot denotes the centroid of the displaced pixels. **d** The uncertainty ellipse region, in magenta, overlaps the MLO lesion in the CC/MLO overlay. **e** The uncertainty ellipse, in yellow, with a red centroid marker shown. **f** A depiction of the area of an uncertainty ellipse, $A_{el}$, and the distance, *s*, between the ellipse centroid and the MLO lesion centroid

#### Training

The networks were implemented in MATLAB^®^. The base architectures representing Networks 1, 2, and 3, depicted in Fig. 1, were used for training. (The ensembles are formed during inference and are applied to test data, as noted in Section “Network Ensembles”.) Training was configured using the Adam optimizer, with an initial learning rate of 0.001 and random weight initialization [44]. Around 50 to 100 epochs were used for training, with a mini-batch size of 32. For the dual-path architectures, one unique training step involved delaying the update of the weights in the variance-related branch until the deformation field is roughly established. This was found to facilitate more stability in the training process [24].

#### Ensemble Configuration

In order to facilitate the construction of ensembles for use at test time, each architecture was trained 15 times, with random weight initializations, and this was done for each data set. To test the performance of different-sized ensembles, seven sizes of ensembles were evaluated, $K = 3,5,7,9,11,13,15,$ in addition to the single networks, $K=1$. Then, for each ensemble size, except for $K = 1$ and $K = 15$, 100 combinations of size *K* ensembles were constructed using the 15 models and applied to the test data. For example, for ensemble size $K=3$, 100 combinations from a total of ${15\atopwithdelims ()3}$ combinations were evaluated.

#### Performance Metrics

Several metrics are utilized to assess performance. The first uses the distance between the centroid of the displaced pixels emanating from the CC lesion ground truth region and the centroid of the MLO lesion ground truth. This is depicted in Fig. 5c. The performance metric is the average of the distances measured for all of the test image pairs.

The second metric involves the use of the Mahalanobis distance [45] as a measure of how close the distribution represented by the uncertainty ellipse is to the MLO lesion centroid. Mahalanobis distance is used for uncertainty estimation in some applications, including for outlier detection, and it represents a normalized, unitless, distance between a point and the mean of a distribution, based on the distribution’s covariance [46, 47].

The third metric involves the use of $95\%$ confidence ellipses that are generated from the covariance estimates (cf. (18)). The confidence ellipses are primarily intended for use as uncertainty measures. If the ellipse is small, it indicates low uncertainty (i.e., high confidence) in the registration mapping, whereas a large ellipse indicates high uncertainty in the mapping. However, we also experiment with the use of the confidence ellipses as a measure of registration success, based on whether or not the $95\%$ ellipses intersect with the target MLO lesion. Referring to Fig. 5d, the registration is deemed successful, if the confidence (i.e., uncertainty) ellipse overlaps the ground truth region for the MLO lesion. This additional use of confidence ellipses for purposes of “containment” has also been reported in other domains [48]. Further, it is supported by the use of error ellipses for the purpose of assessing spatial positioning accuracy [49].

While the first three metrics are geared toward assessing the accuracy and uncertainty of the registration mappings, the fourth metric is employed to give indication of the quality (cf. Section “Related Works”) of the uncertainty ellipses. For each CC and MLO test pair, two quantities are compared as illustrated in Fig. 5f: the area of the resulting uncertainty ellipse, $A_{el}$, and the distance, *s*, between the ellipse centroid and the MLO lesion centroid. The latter is comparable to the aforementioned distance metric (the closeness of the centroid of the displaced CC pixels to the MLO lesion centroid). An ideal registration result would render both $A_{el}$ and *s* to be relatively small. Using all of the CC and MLO test pairs, the correlation between $A_{el}$ and *s* values is computed using the Pearson correlation coefficient [50]. A high correlation indicates that the uncertainty ellipse size increases with greater mis-registration, which is a desired uncertainty characteristic. While, conceptually, uncertainty is generally considered independent of a system’s accuracy [25], for our application domain of CC/MLO lesion registration, we find that uncertainties that give indication to accuracy can be quite useful. As previously noted, uncertainty is also used in other applications for assessing accuracy [49].

Sensitivity and specificity [51] measures are also subsequently computed in one experiment involving a lesion detection application. Further, for all comparative measures between the three ensemble architectures, statistical significance was assessed for each ensemble size, *K*, using one-way analysis of variance (ANOVA) which yields an ANOVA-based *P*-value  [52].

**Fig. 6:**
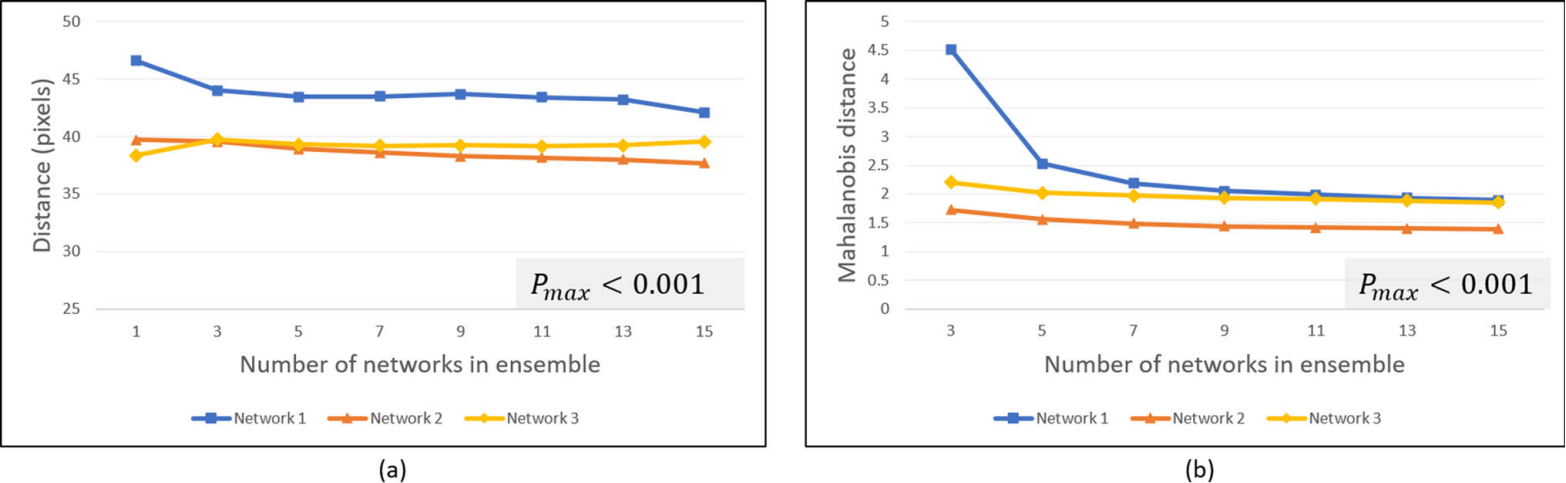
Synthetic data: **a** Average distance between displaced pixels and MLO lesion centroid and **b** median Mahalanobis distance between MLO lesion centroid and the distribution represented by the resulting output covariance matrix for the CC lesion mapping

**Fig. 7:**
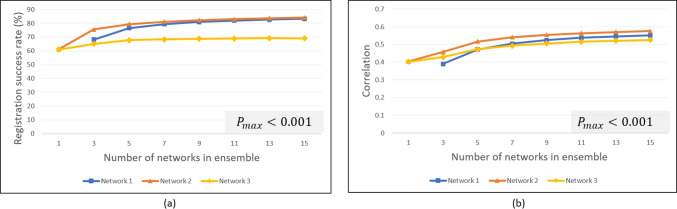
Synthetic data: **a** Ellipse containment-based registration success rates and **b** correlation between ellipse area and closeness of displaced pixels to target lesion

### Results on Synthetic Data

#### Registration Accuracy by Displacement Distance

Figure 6a shows the performance of the ensembles in terms of the closeness of the displaced pixels to the MLO lesion centroid, based on the distance metric discussed in Section “Performance Metrics”. For each ensemble size *K*, the average of the distance values yielded from the 100 combinations (cf. Section “Ensemble Configuration”) (except for $K=1$ and $K=15$) is computed. Hence, in Fig. 6a, the *x*-axis represents the size *K* of the ensemble, and the *y*-axis represents the average distance between the displaced CC lesion pixels and the MLO lesion centroid. The three curves represent the performance for the three networks. It can be seen that ensembles based on Networks 2 and 3 displace the CC lesion pixels closer to the MLO lesion than Network 1. Further, for Networks 1 and 2, a slight improvement in the displacement-to-target lesion proximity occurs as the ensemble size increases.

**Fig. 8:**
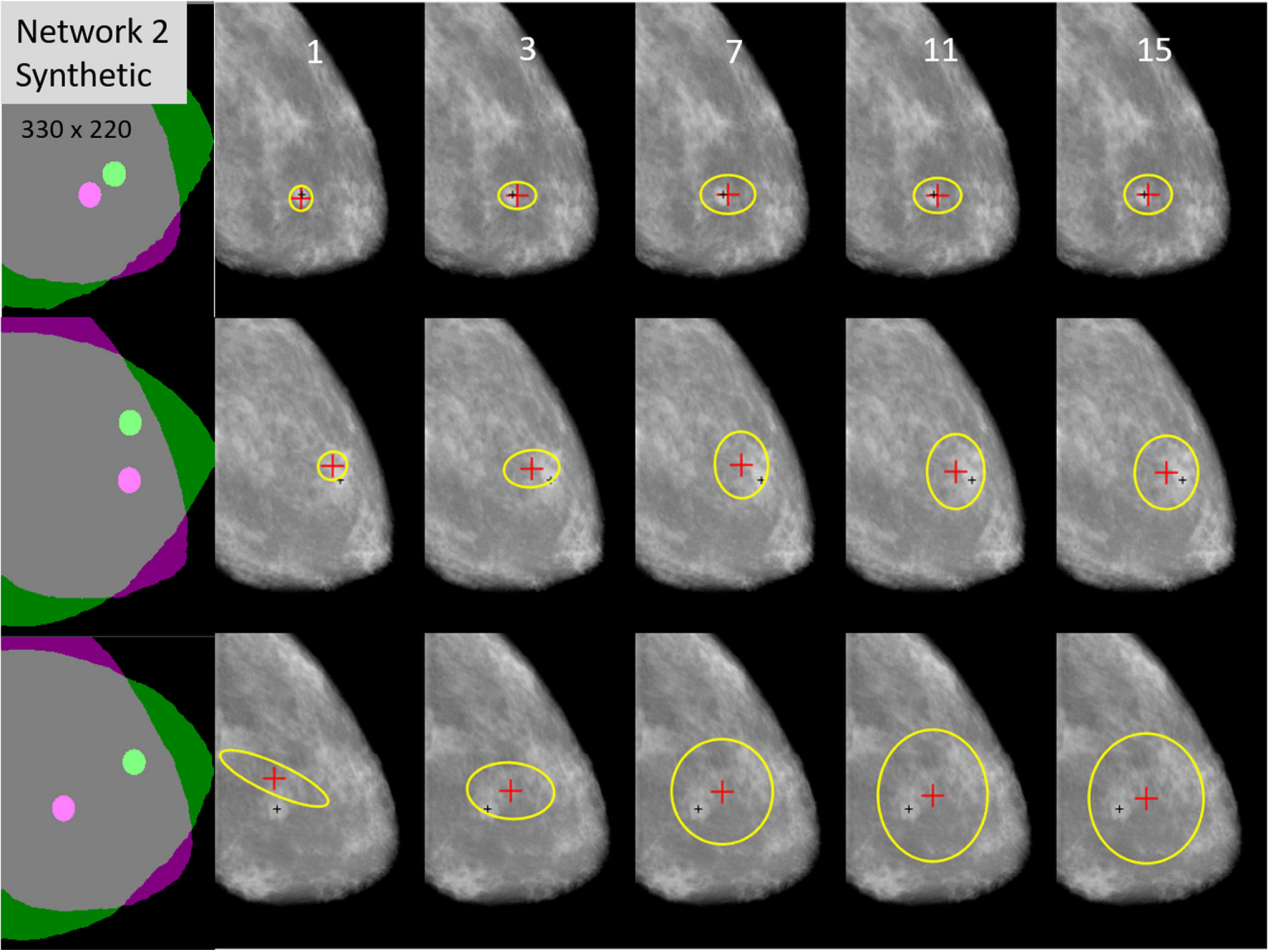
Synthetic data: example uncertainty ellipses from ensembles of different sizes, using Network 2

Figure 6b plots the Mahalanobis distance measure for the ensemble-based displaced pixel distributions for the synthetic data, in further comparing the registration displacement performance. For each ensemble size *K*, the median of the Mahalanobis distance values yielded for the 100 combinations (again, except for $K=1$ and $K=15$) is computed. (The median is chosen, versus the average, due to the effects of outliers for this measure.) It can be seen that the Network 2-based ensembles yield the smallest Mahalanobis distance, which means that the registration,  on average, mapped the distribution of CC lesion pixels closer to the MLO lesion centroid, similar to the observation from Fig. 6a. Also, similar to Fig. 6a, the displacement performance improves (i.e., lower Mahalanobis distance) for each network as ensemble size increases. In Fig. 6 a and b, ANOVA-based *P*-values for each ensemble size, *K*, were relatively low, $P_{k\in K} < (\alpha == 0.05)$ (denoted by the $P_{\text {max}}$ expression on each plot), which indicates statistical significance [52].

#### Ellipse Containment-Based Success Rates and Uncertainty Quality

Figure 7a shows the average registration success rates yielded by ensembles of different sizes using the $95\%$ ellipse containment-based criteria. The *x*-axis again represents the ensemble size *K*, and the *y*-axis represents the average success rate achieved from among the 100 combinations (cf. Section “Ensemble Configuration”) of networks evaluated for that ensemble size. As shown, Networks 1 and 2 yield comparatively higher average success rates than Network 3, with Network 2 yielding the highest average, $84.2\%$, at ensemble size $K=15$. The absolute highest success rate among the 100 Network 2 combinations was $85.4\%$ (not shown) at $K=11$, which was also the highest across all combinations and ensemble sizes from the three networks. We note that for Network 1, there is no ellipse-based success rate shown for $K=1$ since Network 1 does not generate variance estimates, except through an ensemble.

Figure 7b shows values for the fourth performance metric, the correlation between the uncertainty ellipse area and the closeness of the displaced pixels to the MLO lesion, which we use to assess the quality of the uncertainty estimates (cf. Section “Performance Metrics”). For each ensemble size, the average correlation achieved (from among the combinations of networks evaluated at that size) is shown. The correlation values (thus, the quality of the uncertainties) increase with ensemble size. Network 2 consistently yields the highest performance, with correlations approaching 0.6 (moderate correlation [50]).

Figures 6 and 7 demonstrate the superiority of ensembles (i.e., $K > 1$) over single networks and further show a trend of increasing performance as ensemble size increases (with the exception of Network 3 for the displacement measure in Fig. 6a). Network 2 yielded the highest performance for each metric. Analysis revealed that in addition to overall better registration accuracy (cf. Fig. 6) and better correlation between ellipse size and registration accuracy (i.e., quality of uncertainty estimates) (cf. Fig. 7b), Network 2 also tended to generate larger ellipses, which helps account for the higher ellipse containment-based success rates (cf. Fig. 7a).

**Fig. 9:**
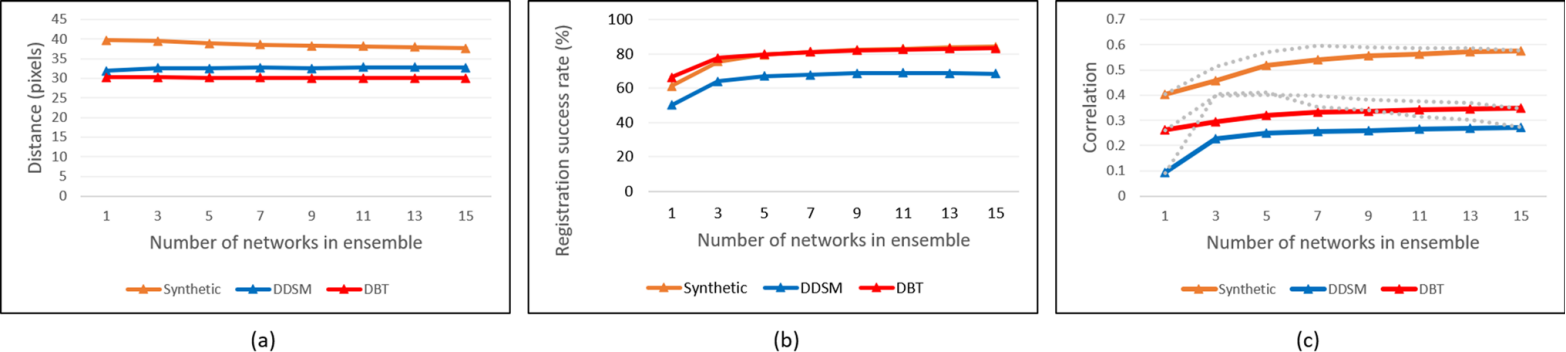
Synthetic and real data: **a** Average distance between displaced pixels and MLO lesion centroid, **b** ellipse containment-based registration success rates, and **c** correlation between ellipse area and closeness of displaced pixels to target lesion. Absolute maximum values yielded from among the 100 combinations of runs, for each *K*, shown as dotted lines (*P*-value $<0.001$ for all ensemble sizes *K* in each plot)

#### Visual Examples

Figure 8 shows examples of uncertainty ellipses for different-sized ensembles using Network 2. Three CC/MLO lesion cases are represented in the left-most column, each by an overlay of the CC and the MLO mask, highlighting the locations of the lesions in green for CC and in magenta for MLO, respectively. The remaining columns represent results from different ensemble sizes, as denoted at the top of each column. The yellow ellipses are based on a $95\%$ confidence value, with the red cross representing the ellipse centroid.

In the top row, involving the case in which the lesion locations between the two views are fairly close, it can be seen that, for each ensemble size, the ellipses are relatively small and are centered fairly close to the MLO lesion. In the second case (middle row), the lesion locations are more separated. The registration successfully maps the CC lesion to the MLO location, though slightly outside of the visible boundary of the lesion. Correspondingly, the ellipses are slightly larger than those in the top row. In the third case (bottom row), the lesion locations are farther apart. The registration is not quite as accurate as in the top and middle rows; thus, the uncertainty ellipses are larger. However, the network still displaces the CC lesion to the general location of the MLO lesion and the ellipses either encompass or intersect the MLO lesion, except for the $K = 1$ case. Hence, for these examples, the ellipse size visibly correlates with the closeness of the registration mapping to the target lesion location, thus giving an indication to the uncertainty of the registration. A trend of larger ellipse size with increasing ensemble size correlates with the observations in Fig. 7a.

In Fig. 8, a distinction is observed in the bottom row for the ellipse yielded by the single network ($K=1$, second column) compared to those yielded by the ensemble-based networks, $K=3$ through 15. Specifically, the ellipse for $K=1$ shows an obvious correlation in the *x* and *y* directions, despite Network 2 yielding only horizontal and vertical variances, with no correlation estimate. This occurs due to the region-based processing (cf. (18)) that is used for the final covariance estimate. In essence, a weighted mixture of Gaussians with uncorrelated variables can result in a correlated Gaussian.

For the three lesion cases, it is noted that there is an apparent correlation between the accuracy of the registration mapping and the separation distance between the lesions. Indeed, among the test data, there is some degree of such correlation. This is in part due to a higher percentage of cases in the training data in which the relative locations of the lesions are closer between the CC and MLO views. Hence, the model learns to register close-to-moderately spaced lesions better than it does lesions with significant separation between the two views. Yet, again, the uncertainty ellipses tend to reflect the accuracy of the registration.

Additional analysis on the uncertainty estimates is provided in Apps. B and C in the Supplementary Material, where, respectively, the effects of the amount of training data are shown, and the superiority of the use of an ellipse versus a circle is compared.

**Fig. 10:**
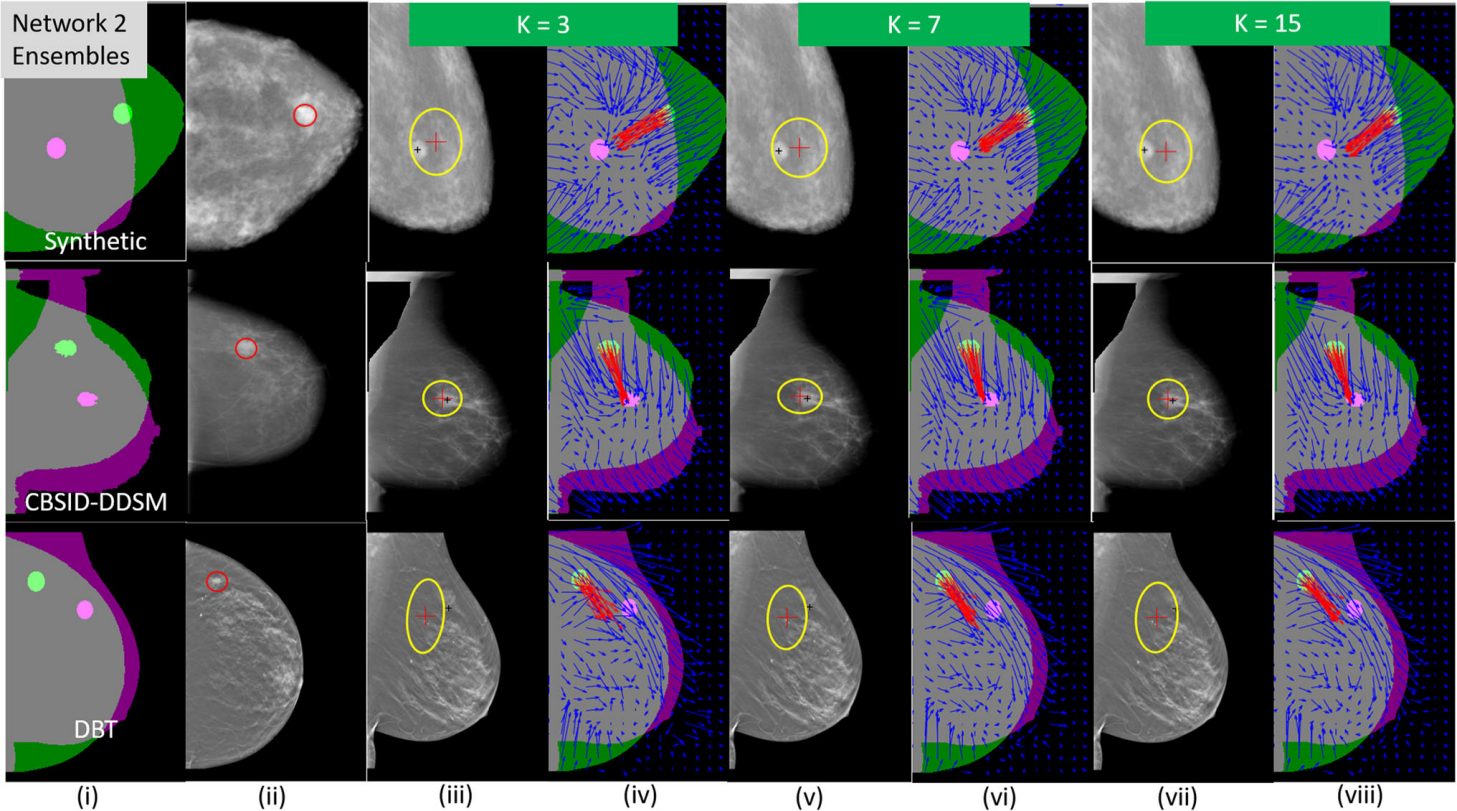
Example uncertainty ellipses with corresponding deformation fields shown for synthetic (top row), digitized scanned film [CBIS-DDSM] (middle row), and slices from DBT (bottom row) X-ray data. Color schemes in cols. **i**, **iii**, **v**, and **vii** are the same as Fig. 8. In col. **ii**, the red circle represents the CC lesion location. In cols. **iv**, **vi**, and **viii**, the deformation vectors, displayed as quiver plots, show the individual pixel displacements. The red vectors represent pixels displaced from the CC lesion position, and the blue vectors emanate from pixels at other locations across the breast

### Results on Real X-ray Data

The set of experiments from Section “Results on Synthetic Data” were repeated for real X-ray data (i.e., CBIS-DDSM and DBT) listed in Table 1. Details of the analysis for CBIS-DDSM are provided App. D in Supplementary Material, where it is revealed that Network 2 continued to yield the best performance. Results with DBT data were not as consistent, and it is believed this was due to overfitting, given the limited DBT training data counts (cf. Table 1). However, in subsequent discussion, we show that the DBT-based models can indeed be effective, despite low data counts. Moreover, analysis revealed that models trained using a 1 : 1 mixture of the synthetic and CBIS-DDSM X-ray data performed better than models trained solely with real X-ray data in tests with the CBIS-DDSM as well as the DBT data. In this section, we show performance comparisons for the mixed data-based models, tested on real X-ray data, along with the performance from the synthetic data.

#### Performance Comparison: Synthetic and Real

Figure 9a compares the performance for the Synthetic, CBIS-DDSM, and DBT-based of the Network 2-based ensembles using the same displacement distance metric as Fig. 6a. For the real data sets, Network 2’s average displacement for the CC pixels is closer to the MLO centroid than was the case for the Synthetic data. While numerous factors could affect the displacement trends such as quantity of training data, lesion sizes, and tissue characteristics, it is noteworthy that the performance is fairly consistent across ensemble sizes for the real X-ray data.

Figure 9b shows that the ellipse-based success rates for the DBT data (when trained using a mixed synthetic/DDSM model) are comparable to that of the synthetic data, though for the CBIS-DDSM data it is approximately 10 percentage points lower. Similar to the case for the synthetic data, the ellipse-based success rates are higher for the ensembles versus a single network, $K=1$.

Figure 9c shows that, in terms of the uncertainty quality, the performance with the real data sets is not as high as that of the synthetic data. The highest average correlation value for the synthetic models is 0.576 at $K=15$ (absolute max. of 0.596, at $K=7$) across all combinations of runs. While the average values for the real data sets are noticeably lower, it was found that the absolute maximum correlation achieved from among the combinations of runs was much higher at 0.41 and 0.4 for DDSM and DBT test data, respectively.

**Fig. 11:**
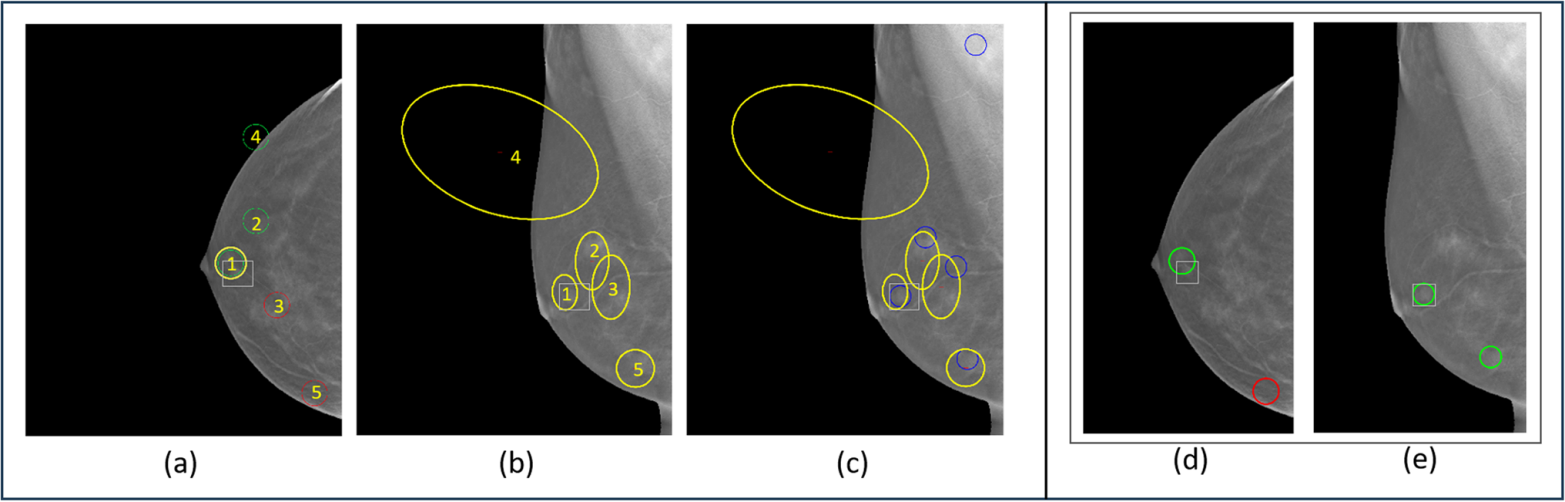
Illustration of how registration uncertainty ellipses can be used for removing false alarm detects yielded by lesion detectors in both the CC and MLO views. The ensemble size was $K=15$. **a** Five candidate detects in the CC view, **b** $95\%$ confidence ellipses at the CC projection locations in the MLO view, **c** overlay of uncertainty ellipses with the MLO candidate detects (blue circles), and **d** and **e** CC and MLO detects that remain when only mappings that overlap with the two smallest ellipses are considered

#### Additional Examples and Deformation Fields

Figure 10 shows additional registration results, along with the corresponding deformation fields for the synthetic test data (top row), CBIS-DDSM test data (middle row), and DBT test data (bottom row). For both the CBIS-DDSM and DBT test data results, the mixed synthetic/CBIS-DDSM-based training models were used given their superior performance on our real X-ray data sets as discussed in Section “Results on Real X-ray Data”. For DBT data, individual slices (2D images) which intersected the lesions were used as test data.

For the examples in Fig. 10, three ensemble sizes are represented, $K=3,7,$ and 15. The deformation fields generated by the ensembles are shown on the CC/MLO mask overlays, where vectors emanating from the CC lesions are shown in red. For the cases with the synthetic and CBIS-DSSM test data (the top and middle rows), the registration maps the lesion fairly close to the MLO lesion location. The red deformation vectors visibly show this, and the uncertainty ellipses generally contain the target MLO lesions. For the DBT case, the red deformation vectors also show that the displacement is generally in the neighborhood of the MLO region, though they are not as aligned with the direction of the MLO lesion. Still, the uncertainty ellipses intersect the MLO lesion. As Fig. 10 shows, based on both the uncertainty ellipses and the deformation fields, our CNN architectures indeed show promise for providing mappings for lesion locations between the CC and MLO views, with indications of confidence, that can be useful for a clinician.

### Use of Uncertainty Estimates for Reducing False Alarms

In a final experiment, the utility of the uncertainty estimates, using DBT-based models, is further illustrated in an application for removing false alarms generated by a lesion detector. In Ref. [53], our original, non-uncertainty-based registration network was used as part of a multi-view, multi-modality fusion-based system which performed lesion detection and diagnosis. The lesion detector in the system individually processed the CC and MLO views. The detector was configured to produce 5 detects in CC and MLO, each, for all test cases. A total of 20 test cases were used, and each case involved a single lesion, which was present in both the CC and MLO views.

To test the utility of the uncertainty-based networks in this application, the Network 2-based ensembles are employed. The ensembles are subjected to the same 2280 DBT training pairs (including augmentations) and the 20 test pairs from Ref. [53] (cf. Table 1). Different sizes of ensembles were tested, similar to the scheme used in Section “Results on Synthetic Data”.

#### Performance Illustration

Figure 11 illustrates the utility of the uncertainty-based registration in one of the test cases (using size $K=15$ ensemble). Figure 11a shows a single slice from the CC view of a DBT cube, which typically involves 60 to 90 slices. Five candidate detects from the lesion detector are shown, numbered 1 through 5. The one true lesion is denoted by the white square, and the lesion has been detected (detect 1). The red or green circles represent malignant or benign predictions, respectively, assigned by the lesion detectors.

Figure 11b shows the $95\%$ confidence ellipses in the MLO view, where the corresponding numbered CC detects are mapped. Thus, CC detect 1 is mapped near the lesion location in the MLO view, as desired. It is also observable that this ellipse is the smallest of the ellipses in Fig. 11b. Since the other ellipses, 2–5, correspond to detects that were not at the lesion location in the CC view, and given that the CNN is trained to register lesion tissue versus other tissue, the larger ellipses are understandable for these detect mappings. In essence, the network is more certain about the mapping between the CC lesion-detect and MLO lesion-detect, than it is for the mappings between the non-lesion CC detects and their corresponding MLO locations. Such a characteristic is exactly what is anticipated for this application.

Figure 11c shows an overlay of both the uncertainty ellipses and the MLO candidate lesion detects (blue circles). It is further observed that the size of the ellipses appears to correlate with the closeness of their centroid to a candidate detect.

Finally, Fig. 11 d and e show the result of removing false alarm detects based on a convention in which only registered MLO detects (and corresponding CC detects) that are overlapped by the two smallest ellipses were maintained. Detects that were exclusively overlapped by larger ellipses, or not overlapped at all, are rejected. In essence, Fig. 11 d and e can serve as the more interpretable outputs for a clinician. There are only two candidate detects per view, with one detect in each view being the actual lesion.

**Table 2:** Comparison of lesion detector and uncertainty-based registration detection performance

Performance	Lesion detector	Uncertainty/registration-based		
measures		detect matching ^1^		
		4 smallest	3 smallest	2 smallest
		ellipses	ellipses	ellipses
Sensitivity (%)	95	95	80	70
Specificity (%)	29	54	64	73
Number of false				
alarms per image pair	3.55	2.30	1.80	1.35

^1^Based on the intersection of the $95\%$ confidence ellipses with the MLO lesions

As Table 2 shows, for all 20 test cases, filtering detects based on the registration uncertainty ellipse size can indeed improve lesion detector’s specificity, without sacrificing sensitivity [51]. (Here, sensitivity and specificity are applied to the correct or incorrect mapping of lesions, regardless of diagnosis.) App. F in the Supplementary Material shows additional statistical trends that demonstrate the utility of the method of reducing false alarms. Further, a case is shown in which the uncertainty ellipses can help detect a lesion that was missed by a lesion detector.

## Discussion

Overall, the experiments showed the significant potential of the proposed techniques for uncertainty estimation for CC/MLO registration. In particular, fair-to-high correlation [50] was exhibited between the uncertainty ellipse size and the closeness of the registered lesions.

Clinically, the uncertainty estimates render automated tools more useful for radiologists in supporting the routine task of establishing CC/MLO lesion correspondence. The uncertainty estimates offer the clinicians a measure of how much the provided CC/MLO mappings can be “trusted.” This can, in turn, aid the clinicians’ decision on whether additional imaging may be needed, reducing diagnosis time and cost. The use of the proposed registration network with uncertainty estimates for mitigating false alarms in an automated lesion detection system is concrete evidence on the positive impact of the proposed methods.

There are several limitations in our experiments. One is limited training data, a common challenge in mammography machine learning research given the lack of publicly available data sets with ground truth. Significant additional training data would allow for more robust models and thorough characterization of the registration uncertainty performance for different lesion and tissue types. For instance, additional performance metrics that account for lesion size, breast density, diagnosis, and other factors could be considered with more extensive and varied data sets. Our results from augmenting synthetic and real training data show some promise as a means for helping to address this challenge.

In terms of network design, an area for further assessment is potential CNN receptive field-of-view constraints, which could affect registration performance in cases where the lesion location is widely separated between the two views. We conducted very limited research in this area using deeper networks, yet our findings indicated that larger quantities of data would be needed for training these. Also, a limitation of the general DBR-based uncertainty approach is that in untruthed regions of breast images, the uncertainties may not be well modelled. However, as was also emphasized in Ref. [8], the objective of the registration is for the mapping of lesions, and there is generally a lack of truth information for other parts of the images to support accuracy assessment.

Our results also did not factor in geometrical characteristics of the breast images, such as relative distance from nipple between the CC and MLO views. We did experiment with several geometrical relationships; however, at most, only marginal improvements for uncertainty characterization were observed. In short, very little correlation was observed between geometrical relations and the distance between the displaced CC lesion pixels and the MLO lesion. Future research, with significantly increased data sets, could help reveal ways of further leveraging such characteristics, that is, in the absence of additional information such as 3D models. This may also result in improvements to the uncertainty estimates.

## Conclusions

Deep ensemble-based techniques for providing uncertainty estimates for a deformation field-based CNN technique for registering lesion locations between the CC and MLO mammographic X-ray image views have been developed. Several architectural variations were experimented with. The techniques were tested using both synthetic and real X-ray data, including slices from DBT data. Several metrics were used to assess performance, including visual analysis. The results show that the ensemble-based approaches can provide indications of uncertainty for the CC/MLO lesion mappings. Further, in a CAD-based lesion detection experiment, the techniques demonstrated the ability to reduce CAD false alarm detects, resulting in an $86\%$ improvement in specificity, while maintaining a $95\%$ sensitivity level, using a limited data set. Therefore, the techniques show promise for aiding clinicians with the routine task of establishing CC/MLO lesion correspondence, by facilitating not only automated registration, but confidence estimates. Future research paths would include evaluation with significantly larger, and varied, data sets and also exploring additional means for utilizing geometrical characteristics of the mammography images.

## Supplementary Information

Below is the link to the electronic supplementary material.

Supplementary file 1 (pdf 2818 KB)

## Data Availability

The synthetic X-ray data utilized in this study can be requested from the author at wwalton1@umbc.edu. The curated subsets of the TCIA DBT and CBIS-DDSM X-ray data sets used in this study are not available; however, the full data sets are publicly available at The Cancer Imaging Archive: https://www.cancerimagingarchive.net The JHM DBT X-ray data used in this study are not publicly available due to privacy, ethical concerns, and IRB regulations. The code used in this study is not available.

## Footnotes

1 As in our prior work, we continue to focus on masses, corresponding to one of the two main categories of breast lesions. Masses and calcifications are commonly treated separately in mammographic image analysis research.

## Declarations

### Disclaimer

Some of the technologies described in this paper may be protected under the US Patent Nos. 11,361,868 and 11,657,497.
