# Supplementary material for: Uncertainty Estimation for Dual View X-ray Mammographic Image Registration Using Deep Ensembles
Source: J Imaging Inform Med. 2024 Sep 23;38(3):1829–45. doi: 10.1007/s10278-024-01244-1 (PMC12092884; doi:10.1007/s10278-024-01244-1)
Supplement: Supplementary file 1 — (pdf 2818 KB) [file 10278_2024_1244_MOESM1_ESM.pdf]

SUPPLEMENTARY MATERIAL - Uncertainty Estimation for Dual View X-ray Mammographic Image Registration using Deep Ensembles

William C. Walton, Seung-Jun Kim

Appendices

This supplement contains additional analysis and experiments.

Appendix A: Deformation Field Patterns

In our prior research, analysis of the deformation fields resulting from the DBR-based network models revealed patterns that appeared to, visually, show some level of correlation with the degree of success of the the registration mappings. In particular, when the deformation vectors emanating from the known CC lesion converged towards a specific location, the mapping was usually on or near the target MLO lesion. Further, when vectors emanating from the CC lesion were more so parallel, in many cases, even if they did not extend to the MLO lesion, they were oriented towards its direction. However, when the vectors were widely scattered, there was usually significantly observable misregistration. Examples of this phenomena are shown in Fig. S1. While there were exceptions to this trend, there was sufficient evidence to support the notion that certain statistical properties of the deformation fields, such as the variance of the displaced CC pixels, could support uncertainty characterization. Further, this observation was considered in assessing if the network could, in addition to a learning the CC/MLO lesion mapping, also learn the uncertainty associated with the mappings.

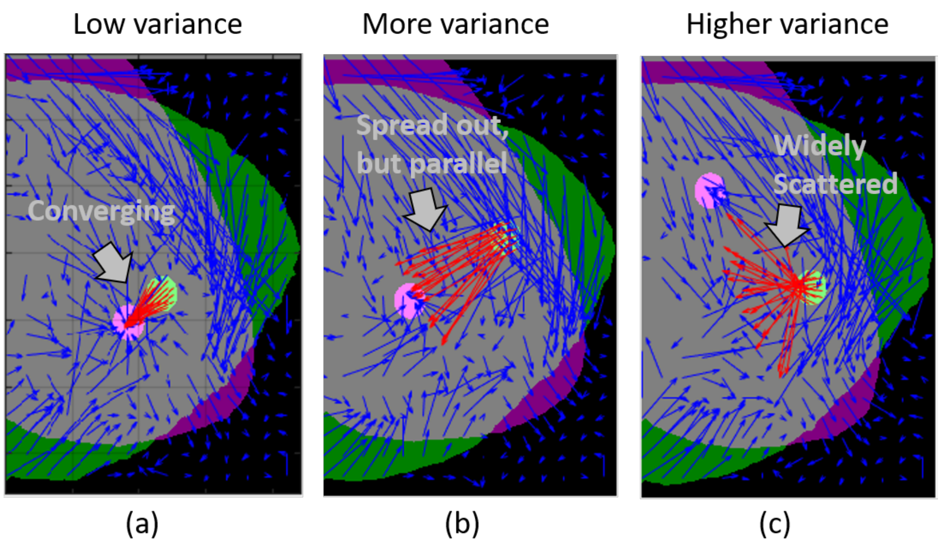

Fig S1 (a) Deformation vectors converging to the center of the MLO lesion. (b) Vectors radiating more so parallel, towards the MLO lesion. (c) Vectors emanating in a widely scattered pattern, mostly away from the MLO lesion.

Appendix B: Performance by Training Data Size

Figs. S2(a) and (b) show the registration displacement accuracy and the ellipse containment-based success rates yielded by ensembles using Network 2 when trained on synthetic data sets consisting of $1000, 2000, 3000, 4000$, and 5000 CC/MLO image pairs respectively. (The results for the 5000 count training data set are those from experiments reported in the main document.) For training data sets of each size, 15 randomly seeded networks were trained to compose ensembles, as was done in the main experiments. Similarly, for each ensemble size K, 100 combinations of networks were selected, except for size $K = 15$. ($K = 1$ was not used for this experiment.) For each ensemble size K the average distance between the displaced CC lesion pixels and the MLO lesion centroid was computed (from all network combinations for that ensemble size bin). The highest ellipse containment-based success rate from multiple combinations of networks at each ensemble size bin was computed.

As shown in Figs. S2(a) and (b), both the the displaced pixel-to-target lesion distance and

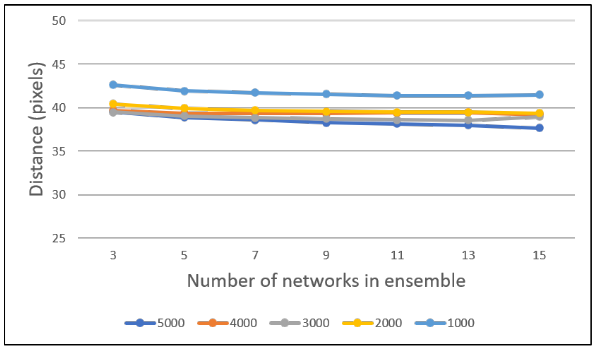

(a)

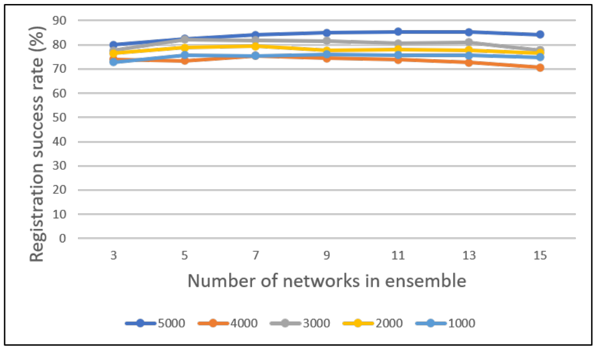

(b)

Fig S2 (a) Average distance between displaced pixels and MLO lesion centroid and (b) Ellipse-based success rates for ensembles trained with on data with counts ranging from 1000 to 5000 CC/MLO pairs.

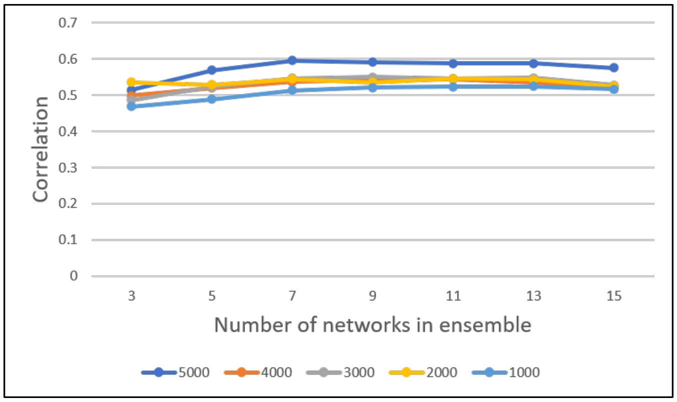

Fig S3 Ellipse-based success rates for ensembles trained with on test data ranging from 1000 to 5000 CC/MLO pairs.

the ellipse containment-based registration success rates were generally better for models trained on larger data counts. That is, the displaced pixel-to-target lesion distance decreased and the ellipse containment-based success rate increased when models were trained with larger data counts. (Oddly, one exception is for the 4000 count training size.)

Fig. S3 shows the highest ellipse area-to-pixel displacement correlation values (i.e., uncertainty estimate quality) yielded by the ensembles for the models trained on the different sized data sets. Here, the correlation values were also generally higher for models trained on larger data sets.

Appendix C: Registration Success with Ellipse versus Circle

Figures S4, S5, and S6 each compare the highest registration success rates yielded by using the 95% confidence ellipse-based criteria compared to that yielded by a circle with the same geometrical area as the ellipse. The results are for Network 2. The ellipse-based uncertainty yields higher registration success rates, which indicates that the covariance estimates generated by the network are useful for orienting an ellipse, at the displaced pixel location, such that the ellipse is more likely to intersect the target MLO lesion, compared to a circle.

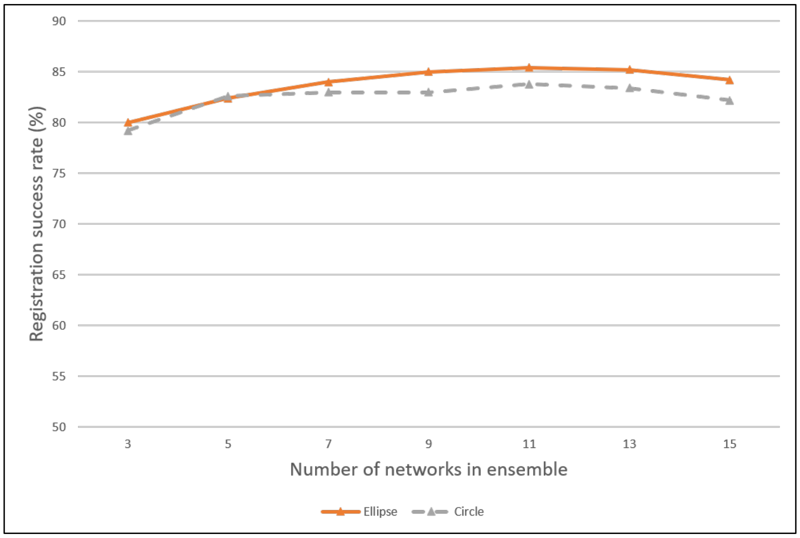

Fig S4 Comparison of the registration success yielded when using a 95% confidence ellipse versus a circle with the same geometrical area (for synthetic data) using Network 2.

Appendix D: Details for CBIS-DDSM Data Results

Two sets of experiments were conducted using the CBIS-DDSM data. The first involved using the designated CBIS-DDSM training and test data as described in Table 1 in the main manuscript.

The second involved augmenting the CBIS-DDSM data with the synthetic data at a $1:1$ ratio. The resulting mixture involves $8,928$ training pairs, versus $4,464$ using solely real X-ray. The same test data, involving 146 pairs of real X-ray data, is used for both experiments.

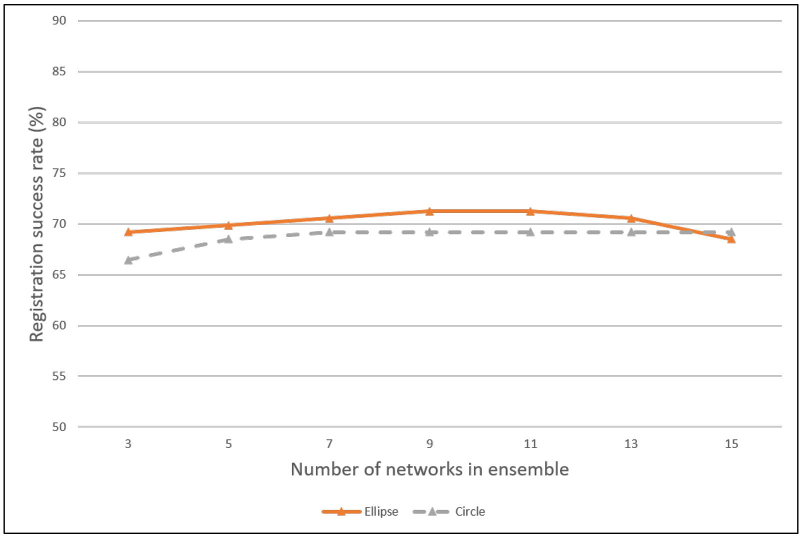

Fig S5 Comparison of the registration success yielded when using a 95% confidence ellipse versus a circle with the same geometrical area (for a mixed real/synthetic model tested on DDSM data) using Network 2.

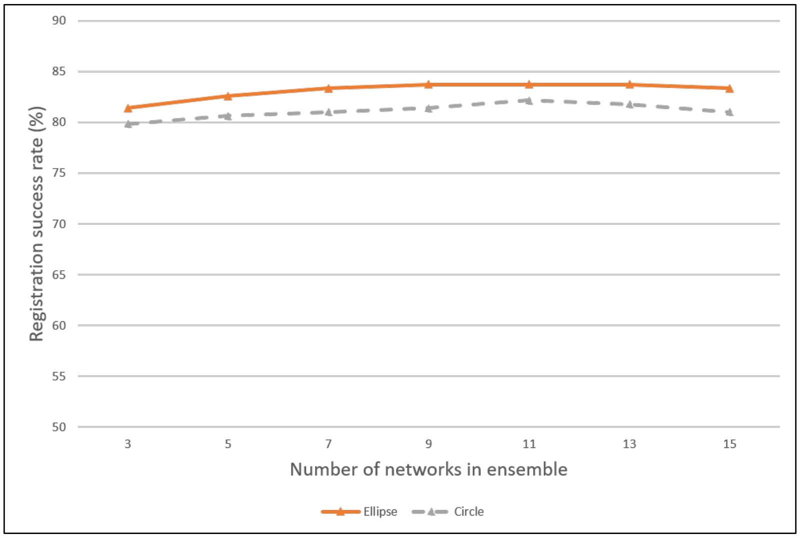

Fig S6 Comparison of the registration success yielded when using a 95% confidence ellipse versus a circle with the same geometrical area (for a mixed real/synthetic model tested on DBT data) using Network 2.

D.1 Registration Accuracy by Displacement Distance

Figs. S7(a)-(d) show the registration displacement performance achieved on the CBIS-DDSM test data. (Lower values are better for this measure.) For the CBIS-DDSM based training models, Fig. S7(a) shows that Network 1 outperformed Networks 2 and 3 in displacing the CC lesion pixels closer to the MLO lesion using a Euclidean approach. However, Fig. S7(c) shows that

with the Mahalanobis-based distance measure, Networks 2 and 3 perform better. Subsequently, it will also be shown that the quality of the uncertainty estimates is not as high for the Network 1 DDSM-trained model.

Fig. S7(b) shows that with the mixed-data training models, the performance between the networks is not as distinct, though Network 2 yields the best performance, with the closest displacement distance of 30.8 pixels at $K = 1$. Fig. S7(d), shows that Network 2 consistently yields the lowest Mahalanobis distance, with values also being lower than those generated by the CBIS-DDSM based models, shown in Fig. S7(c). From Figs. S7(c) and (d), it is also clear that Networks 2 and 3 yield lower Mahalanobis distance values than Network 1 for both CBIS-DDSM and mixed data-based models. In Fig. S7(c), for the CBIS-DDSM based training model, Network 3 slightly outperforms Network 2 for the Mahanobis-based measure, except at $K = 15$.

D.2 Ellipse Containment-based Success Rates and Uncertainty Quality

Fig. S8(a) shows that for the CBIS-DDSM based training models, Networks 2 and 3 yielded higher ellipse-based success rates (i.e., containment) than Network 1. For the mixed-data training models, Fig. S8(b) shows that Network 2 generally yields a somewhat higher ellipse-based registration success rate than Networks 1 and 2. While the higher ellipse-based success rates also indicate higher uncertainty, again, the *quality* of the uncertainty is also important.

Figs. S8(c) and (d) show values for the correlation (i.e., uncertainty quality) between the uncertainty ellipse area and the closeness of the displaced pixels to the MLO lesion for the CBIS-DDSM and the mixed real/synthetic-based training models, respectively, when tested on the DDSM test data. For Networks 2 and 3, it is immediately clear that the correlation values are higher for ensembles based on models trained using the mixture of real and synthetic data, Fig. S8(d), compared

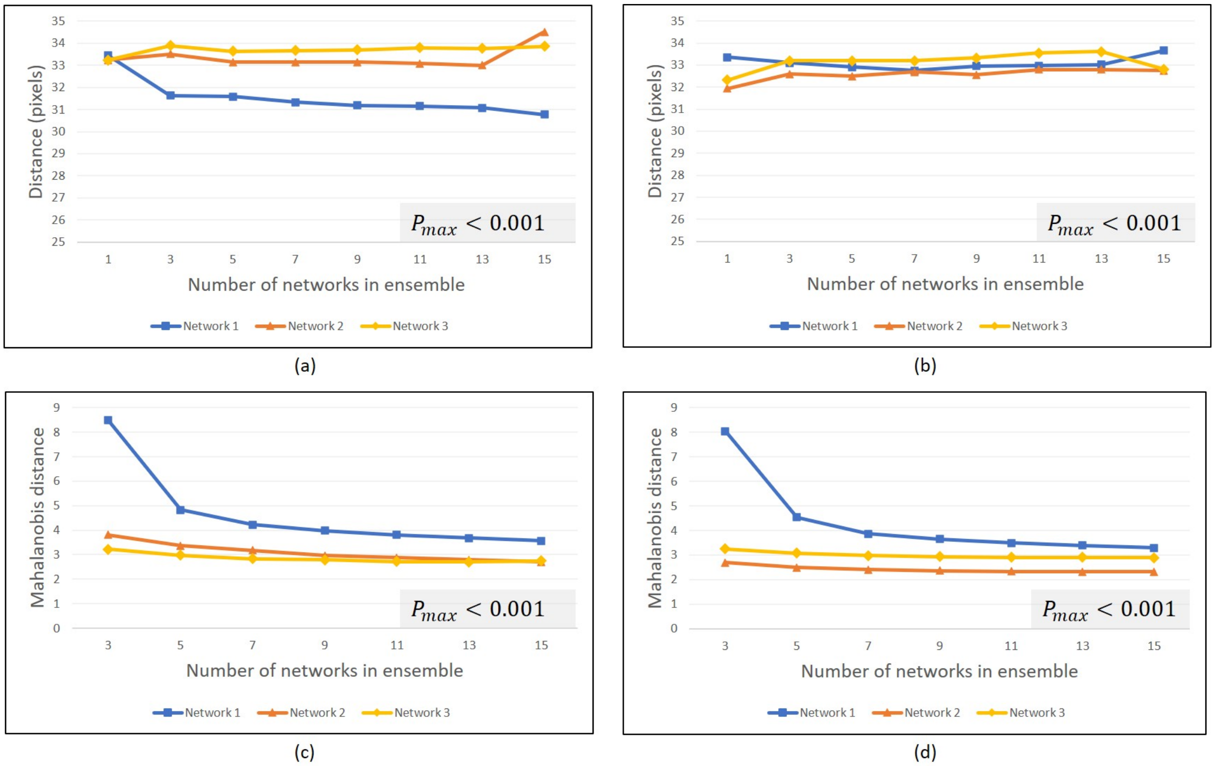

Fig S7 Average distance between displaced pixels and MLO lesion centroid for (a) Model trained on DDSM data and tested on DDSM data and (b) model trained on mixed data and tested on DDSM data. Median Mahalanobis distance between MLO lesion centroid and the distribution represented by the resulting output covariance matrix for the CC lesion mapping for (c) model trained on DDSM data and tested on DDSM data and (d) Model trained on mixed data and tested on DDSM data.

to those trained using only the real data, Fig. S8(c). However, Figs. S8(c) and (d) also reveal that the average correlation values from the CBIS-DDSM and mixed models are not as high as those from the synthetic data. The synthetic models yield average correlation values as high as 0.576 at $K = 15$ (with an absolute max. of 0.596, at $K = 7$ across all combinations of runs).

The average correlation values for the CBIS-DDSM and mixed models (when tested on CBIS-DDSM data) reach 0.093 and 0.273, respectively. However, as also shown in Figs. S8(c) and (d), via the dotted lines, certain combinations of the networks (selected from among the 100 trained models, discussed in the main manuscript) yield significantly higher correlations. The absolute maximums achieved for CBIS-DDSM and mixed models are 0.29 and 0.41, which are both yielded

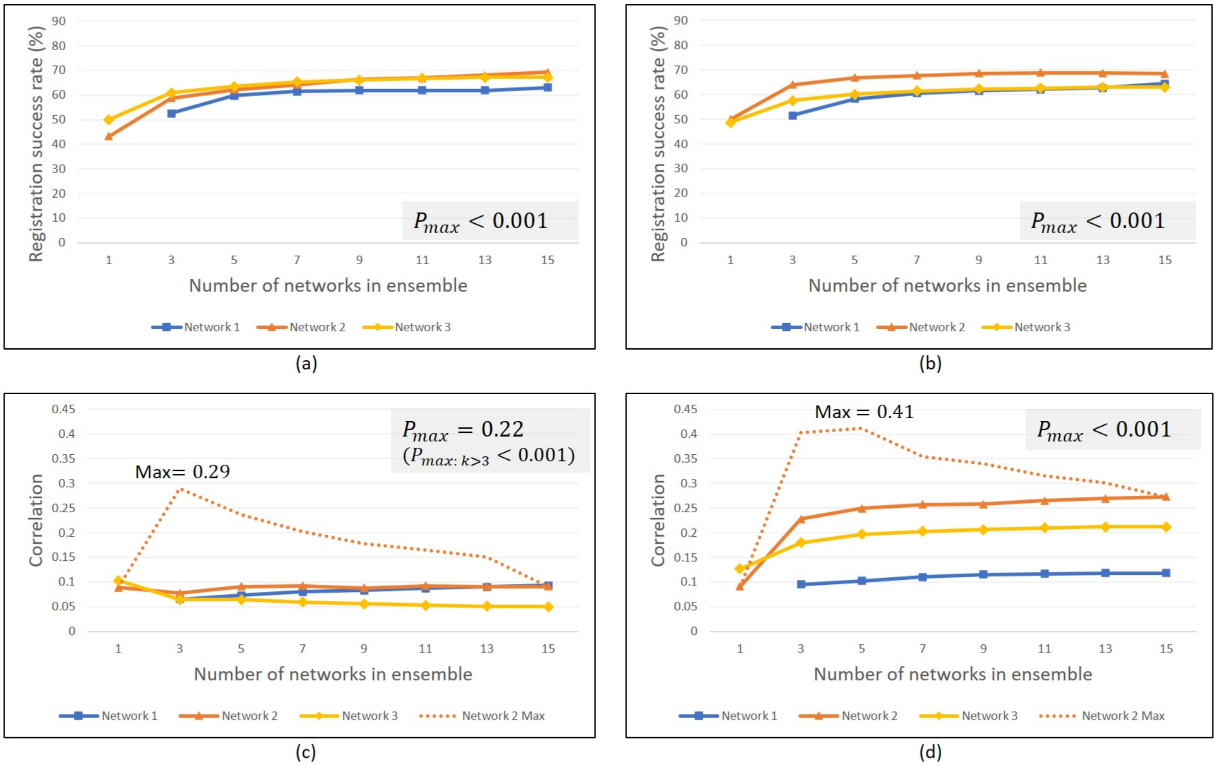

Fig S8 Ellipse containment-based success rates for (a) model trained on DDSM data and tested on DDSM data and (b) Model trained on mixed data and tested on DDSM data. Correlation of ellipse area and closeness of displaced pixels to target lesion (i.e.,uncertainty quality) for (c) model trained on DDSM data and tested on DDSM data and (d) Model trained on mixed data and tested on DDSM data.

by Network 2.

Figs. S9(a) and (b) further reveal the similarity between the ellipse containment-based success rates yieled by the CBIS-DDSM and mixed training models, for Network 2, in contrast to the differences in the uncertainty ellipse area correlation characteristics. For the mixed data-based model, Fig. S9(b), $K = 15$ can be considered the best performing ensemble size since the correlation (i.e, uncertainty quality) is highest at this level, with a fairly consistent ellipse-based success rate for the range of ensemble sizes evaluated.

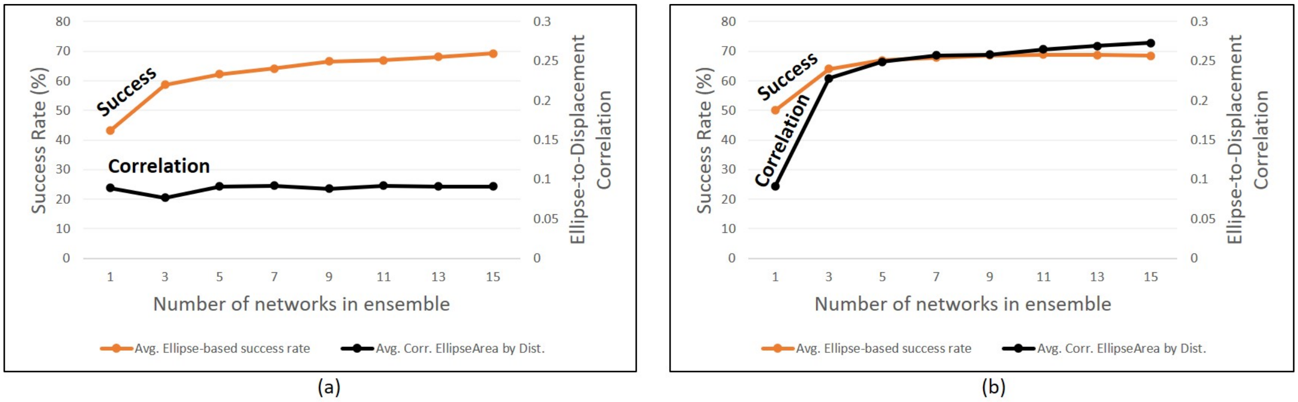

Fig S9 Overlay of registration success rates (left y-axis) and ellipse correlation plots (right y-axis) for Network 2. (a) Model trained on DDSM data and tested on DDSM data and (b) Model trained on mixed data and tested on DDSM data.

D.3 Visual Examples

Fig. S10 shows examples of uncertainty ellipses for different-sized ensembles using Network 2 when trained on the mixed real/synthetic data. Similar to Fig. 8 in the main manuscript, three cases of lesions are shown. In the top row, when not much displacement is required, the registration is very accurate, with a relatively small uncertainty ellipse. For the second case (middle row), the registration mappings are relatively close to the MLO lesion, though not centered on the lesion. The ellipses are correspondingly slightly larger than those in the first case. For the third case (bottom row), the ellipses are centered relatively far from the MLO lesion, and are notably larger than those of cases one and two, thus indicating higher uncertainty for this case, which is appropriate, giving the degree of mis-registration.

Similar to the synthetic data, there is also some observed correlation between lesion location proximity in the two views and registration accuracy. Here again though, this is in part due to the nature of the distribution of lesion locations in the training data. Also, for the CBIS-DDSM data, there was significantly less training data [cf. Table 1 in main manuscript] on which to build robust models, even when augmented with equal-parts of synthetic data, compared to the the amount of

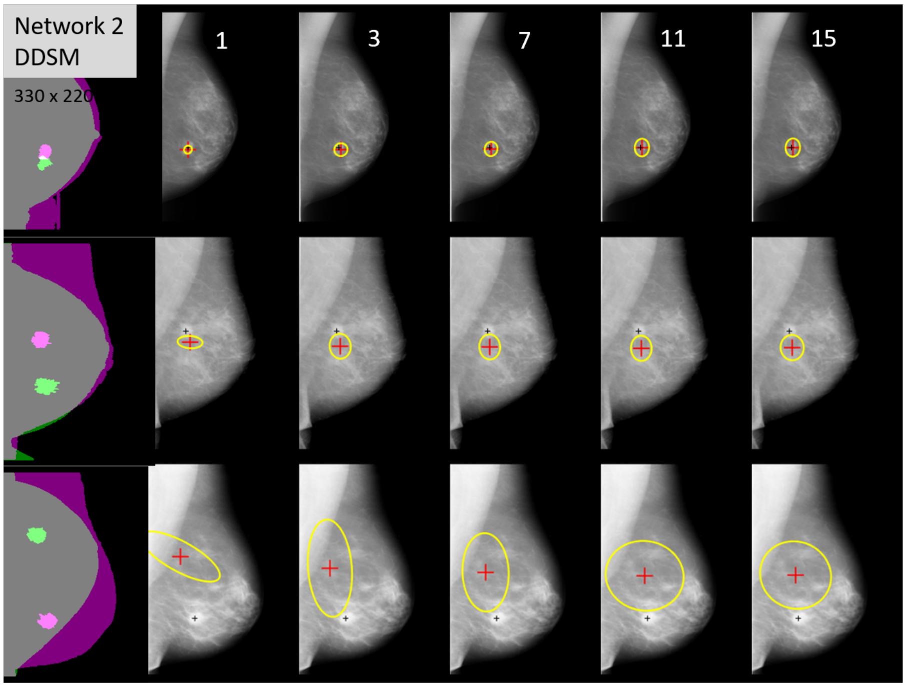

Fig S10 Example uncertainty ellipses from ensembles of different sizes, using Network 2, trained with mixed real CBIS-DDSM/synthetic data.

synthetic training data.

Appendix E: Uncertainty Correlation Analysis

Additional insight on the quality of the uncertainty ellipses can also be gained from scatter plots that show the relation between the ellipse area and the proximity of the displaced CC pixels to MLO lesion centroid for test data pairs. Fig. S11 shows this for models that yielded the highest correlation values from three of the experiments.

A moderate degree of correlation is visible from the plots, which is corroborated by correlation values (shown in the upper right corner of each plot). The significantly low statistical p-values further validate the results. From Fig. S11(a), in particular, it can be observed that with good registration uncertainty, there is generally a lower displaced-to-target lesion separation distance.

However for each model it is also observable, from the red circles, that there are instances

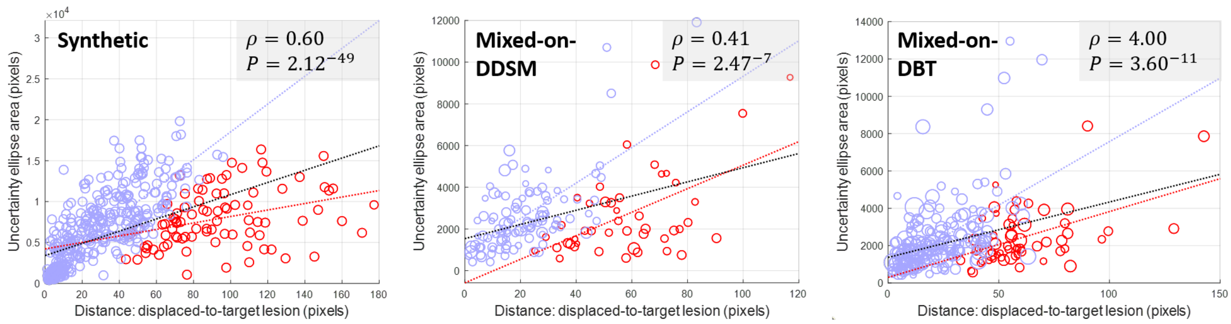

(a) (b) (c)

Fig S11 Scatter plots showing correlation between area of uncertainty ellipses and closeness of displaced pixels to target lesion for (a) synthetic model tested on synthetic data, (b) mixed synthetic/real model tested on CBIS-DDSM data, (c) mixed synthetic/real model tested on DBT data. The circle size relates to the relative size of the lesion. Blue and red represent successful registration and mis-registration, respectively. Blue and red regression lines are also provided for registered and mis-registered points, respectively. The black regression line represents all plotted points.

of relatively small uncertainty ellipses occurring where the displaced CC pixels were not deemed close enough to the MLO lesion to constitute successful registration. While this is in part attributed to some degree of limitation in the robustness of the training models, analysis of the network designs also revealed that there may have been effects from receptive field-of-view constraints in our architecture designs. For example, for cases involving widely separated lesions, limitations in the networks receptive field-of-view may have hampered the networks ability to *sense* both lesion locations.

Appendix F: Uncertainty for Detecting ”Missed” Lesions

Quantitatively, a trend regarding the uncertainty ellipse sizes, in relation to correct lesion-detect matching, is shown is shown Fig. S12. The x-axis represents the ensemble size. The y-axis values represent the median of the areas of the ellipses. As depicted by the two curves, median values are computed for two groups of ellipses. The first group, depicted by the blue curve, involves the subset of CC/MLO detect combinations from the 20 CC/MLO test pairs for which the registration successfully mapped a detect at the CC lesion location to a detect that was at the MLO lesion

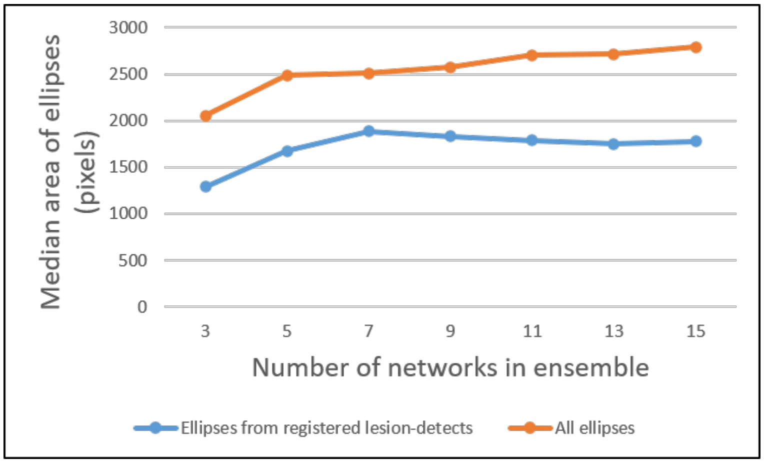

Fig S12 Comparison of the median area for ellipses corresponding to successful CC-to-MLO lesion-detect registration versus the median area of all ellipses.

location, using the criteria discussed in the main manuscript. Note that there could be more than one candidate detect at the location of a lesion in either view. The orange curve represents the median size of all ellipses.

It is immediately clear that ellipses which correspond to successful registration of CC/MLO lesion detects, are generally smaller than median size of all ellipses. The largest difference between the median values occurs at the largest ensemble size, $K = 15$. Here again, this ellipse-size trend is exactly the desired characteristic of the uncertainty ellipses for this application.

Fig. S13 shows an example in which the ensemble-based uncertainties can be useful even when the lesion is missed by the lesion detector in one of the views. Fig. S13(a) shows that in the CC view, detects 1 and 2 are both at the lesion ground truth location. However, as Fig. S13(c) shows, in the MLO view, none of the five candidate MLO detects (in blue) overlap the lesion. Still, as Fig. S13(b) shows, the registration mapped both CC detects 1 and 2 to the location of the lesion in the MLO view. Further, their uncertainty ellipses are dramatically smaller than the large ellipses corresponding to the three CC view false alarm detects and their mappings. Therefore, in this case the registration-mapping uncertainties could be useful for helping to ensure that the actual lesion is

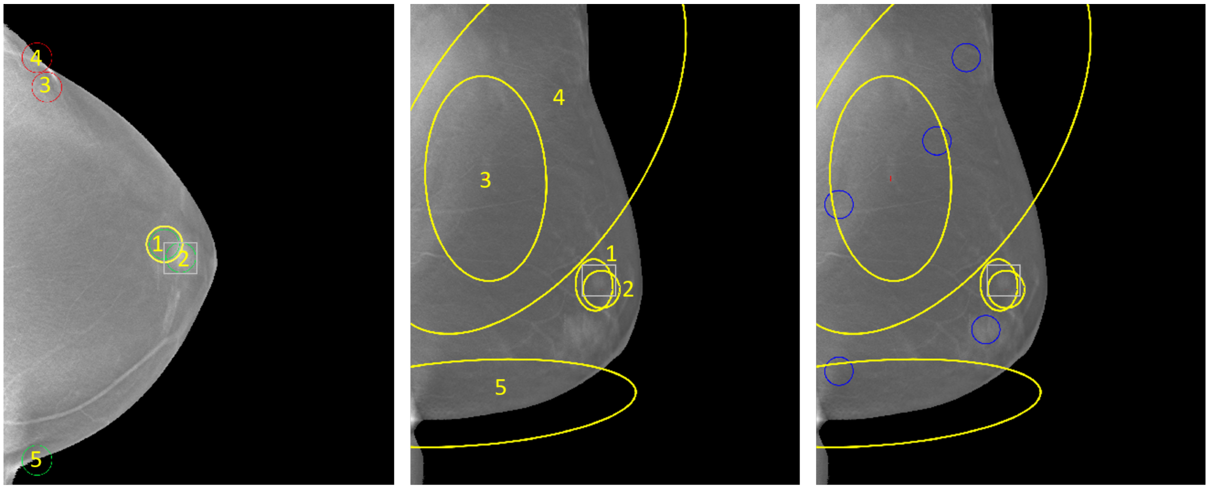

(a) (b) (c)

Fig S13 Example of ensemble-based registration and uncertainty for mapping for case involving a case in which the lesion detector missed the lesion in one view.

detected by the system, even though the lesion detector missed the lesion in one of the views. This shows even more utility for the ensemble-based registration and uncertainty estimation techniques.
